# Exploration of Solid-State *vs* Solution-State Structure in Contact Ion Pair Systems: Synthesis, Characterization, and Solution-State Dynamics of Zinc Diphenyl Phosphate, [Zn{O_2_P(OPh)_2_}_2_], Donor-Base-Supported Complexes

**DOI:** 10.1021/acs.inorgchem.2c03539

**Published:** 2023-03-14

**Authors:** Andrew J. Straiton, James D. Parish, Joshua J. Smith, John P. Lowe, Andrew L. Johnson

**Affiliations:** † Department of Chemistry, University of Bath, Bath BA2 7AY, U.K.; ‡ Milton Hill Business & Technology Centre, Infineum UK Ltd, Milton Hill, Abingdon OX13 6BB, U.K.; § Material and Chemical Characterisation Facility (MC^2^), University of Bath, Bath BA2 7AY, U.K.

## Abstract

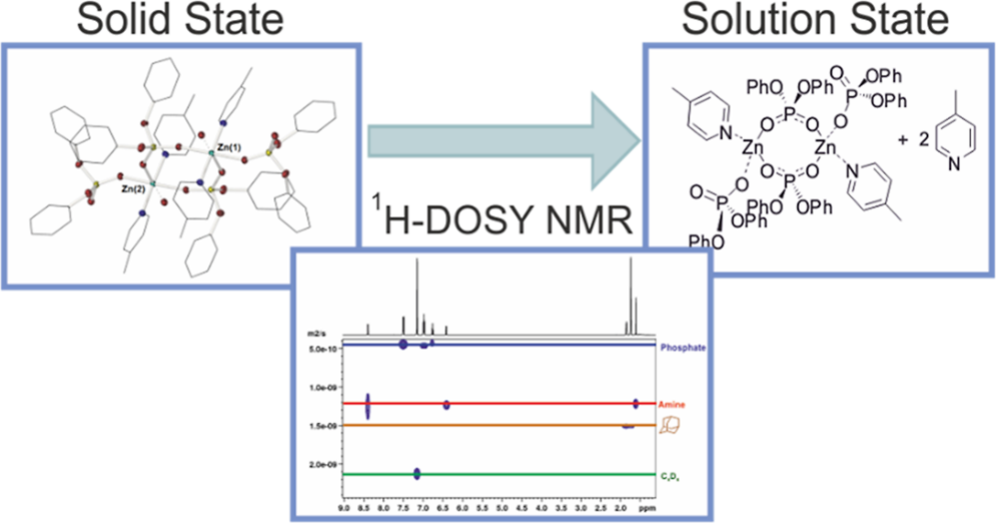

A family of zinc phosphate complexes supported by nitrogen donor-base ligands have been synthesized, and their molecular structures were identified in both the solid (X-ray crystallography) and solution state (DOSY NMR spectroscopy). [Zn{O_2_P(OPh)_2_}_2_]_∞_ (**1**), formed from the reaction of Zn[N(SiMe_3_)_2_]_2_ with HO(O)P(OPh)_2_ coordinates to donor-base ligands, *i.e.*, pyridine (Py), 4-methylpyridine (4-MePy), 2,2-bipyridine (bipy), tetramethylethylenediamine (TMEDA), pentamethyldiethylenetriamine (PMDETA), and 1,3,5-trimethyl-1,3,5-triazacyclohexane (Me_3_-TAC), to produce polymeric 1D structures, [(Py)_2_Zn{O_2_P(OPh)_2_}_2_]_∞_ (**2**) and [(4-MePy)_2_Zn{O_2_P(OPh)_2_}_2_]_∞_ (**3**), the bimetalic systems, [(Bipy)Zn{O_2_P(OPh)_2_}_2_]_2_ (**4**), [(TMEDA)Zn{O_2_P(OPh)_2_}_2_]_2_ (**5**), and [(Me_3_-TAC)Zn{O_2_P(OPh)_2_}_2_]_2_ (**7**), as well as a mono-nuclear zinc bis-diphenylphosphate complex, [(PMDETA)Zn{O_2_P(OPh)_2_}_2_] (**6**). ^1^H NMR DOSY has been used to calculate averaged molecular weights of the species. Studies are consistent with the disassembly of polymeric **3** into the bimetallic species [(Me-Py)_2_·Zn_2_{O_2_P(OPh)_2_}_4_], where the Me-Py ligand is in rapid exchange with free Me-Py in solution. Further ^1^H DOSY NMR studies of **4** and **5** reveal that dissolution of the complex results in a monomer dimer equilibrium, *i.e.*, [(Bipy)Zn{O_2_P(OPh)_2_}_2_]_2_ ⇆ 2[(Bipy)Zn{O_2_P(OPh)_2_}_2_] and [(TMEDA)Zn{O_2_P(OPh)_2_}_2_]_2_ ⇆ 2[(TMEDA)Zn{O_2_P(OPh)_2_}_2_], respectively, in which the equilibria lie toward formation of the monomer. As part of our studies, variable temperature ^1^H DOSY experiments (223 to 313 K) were performed upon **5** in *d*_8_-tol, which allowed us to approximate the enthalpy [Δ*H* = −43.2 kJ mol^–1^ (±3.79)], entropy [Δ*S* = 109 J mol^–1^ K^–1^ (±13.9)], and approximate Gibbs free energy [Δ*G* = 75.6 kJ mol^–1^ (±5.62) at 293 K)] of monomer–dimer equilibria. While complex **6** is shown to maintain its monomeric solid-state structure, ^1^H DOSY experiments of **7** at 298 K reveal two separate normalized diffusion coefficients consistent with the presence of the bimetallic species [(TAC)_2–*x*_Zn_2_{O_2_P(OPh)_2_}_4_], (*x* = 1 or 0) and free TAC ligand.

## Abstract 2

Here, we describe a family of structurally diverse N-donor-supported zinc bis(diphenyl−phosphate) complexes (**1**−**7**), the solid-state structures of which have been determined by single-crystal X-ray diffraction analysis. As part of our study, we have sought to elucidate the solution-state structures of these complexes by ^1^H NMR DOSY experiments, which clearly show ligand rearrangement and redistribution in the solution state.

## Introduction

Single-crystal X-ray diffraction (SCXRD) is one of the most powerful techniques for the structural investigation of metal complexes, providing detailed and accurate data on the structure of these systems. However, SCXRD provides limited information on the nature of the solution-state structures as solid-state structures may not always be retained in the solution state due to an array of solvent interactions and exchange processes. To establish any structure–stability–activity relationships, an intimate knowledge of the speciation and of the most plausible chemical forms in solution is essential as elucidation of the colligative properties in the solution state can have a profound effect upon the understanding of how molecules react.

Several techniques for this exist, for example, small-angle X-ray scattering (SAXS) and nuclear magnetic resonance (NMR) spectroscopy.^1^ While SAXS enables determination of the size and shape of particles in a sample, as well as fingerprinting, it provides less structural chemical information and has size limitations. In contrast, NMR spectroscopy enables the elucidation of the chemical connectivity of a species; however, it can be difficult to distinguish a mixture of complexes in a one-dimensional (1D) spectrum. Diffusion-ordered spectroscopy (DOSY)^2^ has gained increasing importance as a method by which complicated dynamic systems containing discrete molecules/contact ion pairs, as well as solvent-separated ion pairs can be studied. The DOSY experiment seeks to separate NMR signals of different species according to their diffusion coefficients which are sensitive to the size and shape of the molecular species.

Several empirical methods for relating diffusion coefficients to molecular weight (MW) have been proposed; however, no “simple” relationship between diffusion coefficient and molecular weight exists. Recent work by Neufeld and Stalke et al.^3^ developing external calibration curves (ECCs) with normalized diffusion coefficients for alkaline and alkaline-earth metal systems has facilitated the determination of MWs of inorganic compounds via DOSY and provided much more accurate results than previous studies.^4^

As part of our ongoing interest in new molecular precursors for thin-film applications and aerosol-assisted chemical vapor deposition (AA-CVD),^5^ we turned our attention to molecular complexes incorporating organophosphate ligands. In terms of thin-film applications, zinc phosphates have attracted attention from inorganic and material chemists because of their application as a protective layer on metal parts for corrosion resistance and lubrication.^6^ Interest has also been garnered because of their relevance to catalysis,^7^ ion-exchange,^8^ gas storage,^9^ tribochemistry,^10^ nanoparticle formation^11^ as ion hosts for solid-state lasers,^12^ metalloenzymes,^7b,13^ as well as anti-bacterial coatings^14^ and dental cements.^15^

**Figure 1:**
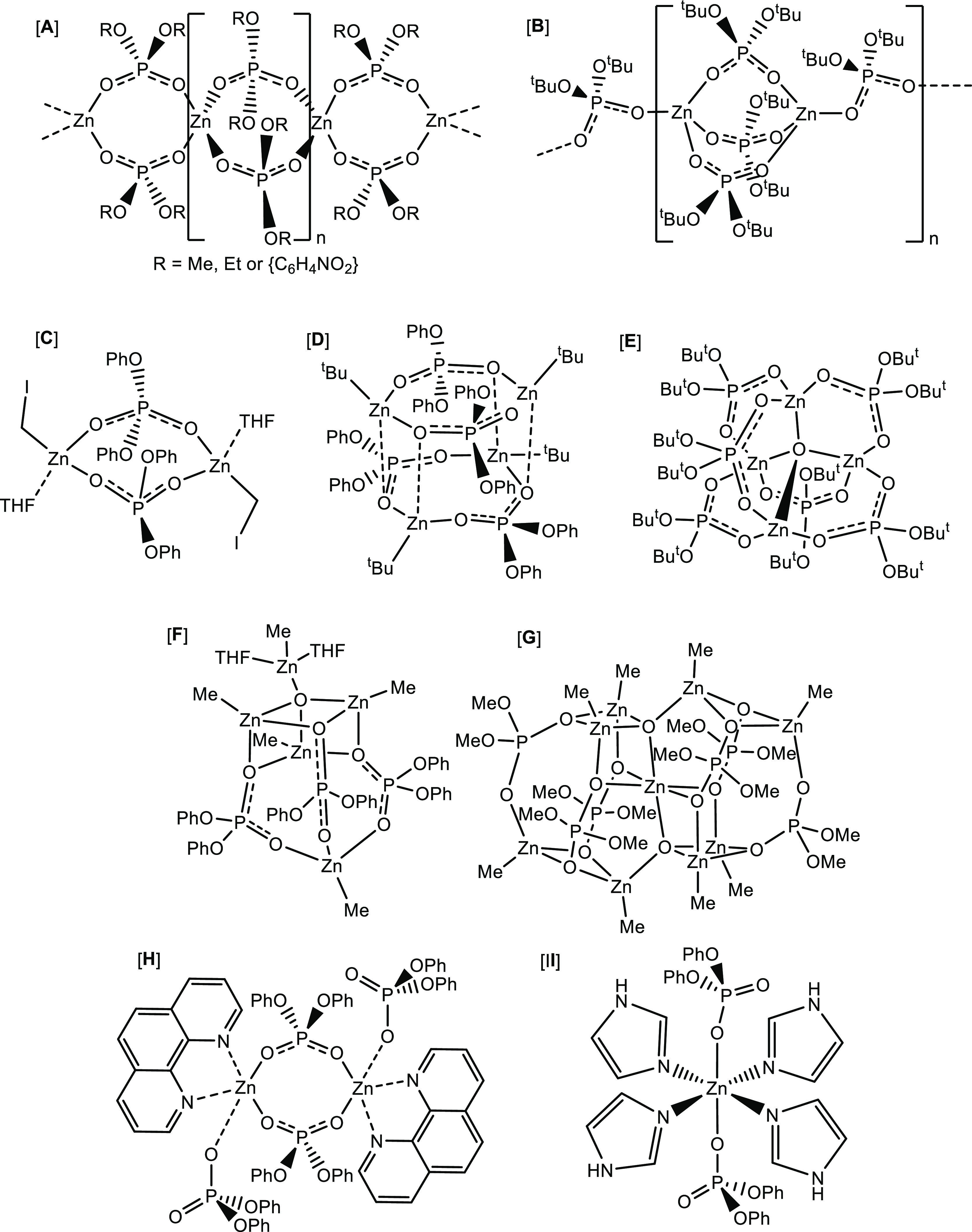
Solid-state molecular structures of selected zinc phosphate systems **A–I**.

Although rational synthetic routes to metallo-phosphates are yet to be firmly established, empirical studies suggest that the structure, nuclearity, and composition of these materials can be varied, and even controlled to some extent, by augmentation of the precursor dialkyl/aryl-phosphates, [(L)_*x*_M{O_2_P(OR)_2_}_*n*_], and mono-alkyl/aryl phosphates [(L)_*x*_M{O_3_P(OR)}_*n*_] (R = aryl or alkyl), by alteration of reaction conditions, or a considered choice of auxiliary donor base ligand (L), which influence precursor aggregation and decomposition temperatures, in addition to decomposition mechanisms.^16^ Examples of known zinc phosphate complexes are shown in Figure 1.^17^

In contrast to zinc phosphates with extended structures, only a limited number of molecular (non-polymeric) zinc phosphate systems have been reported to date,^7d,16d,16e,18,19^ with only a limited number of studies having explored the role of co-ligands in metal phosphate complexes. However, studies on the related zinc O,O′-dialkyldithiophosphate complexes [Zn{S_2_P(OR)_2_}_2_]_2_ (ZDDP) have shown that complexation of the zinc center to a series of simple primary amines results in a range of coordination complexes depending on the amine structure, which in turn has an effect on the physical properties of the resulting material on decomposition.^20^

Studies also suggest that the structures of the molecular metal phosphates are very dependent on the type of metal precursor used, the substituents on the phosphorus atom, as well as the nature of any co-ligands employed for additional stabilization. For example, the reaction of diethylzinc with di-*tert*-butyl phosphate yields the 1D polymer [Zn{O_2_P(O^t^Bu)_2_}_2_]_*n*_, (Figure 1B).^17a^ In contrast, the same ligand reacts with either diethyl zinc, in the presence of water, or with zinc acetate,^19^ to produce the oxido-bridged tetranuclear cluster [Zn_4_(μ^4^-O){O_2_P(O^t^Bu)_2_}_6_] (Figure 1E). Similarly, the reaction of dimethyl zinc with diphenyl phosphate in THF results in the formation of the pentanuclear complex [{MeZn(O_2_P{OPh}_2_)}_3_(Me_2_Zn_2_O)(THF)_2_] (Figure 1F), whereas the reaction of the same reagents in toluene results in the formation of the aggregate [(MeZn{O_2_P(OPh)_2_})_6_(Me_2_Zn_3_O_2_)].^16e^

**Scheme 1:**
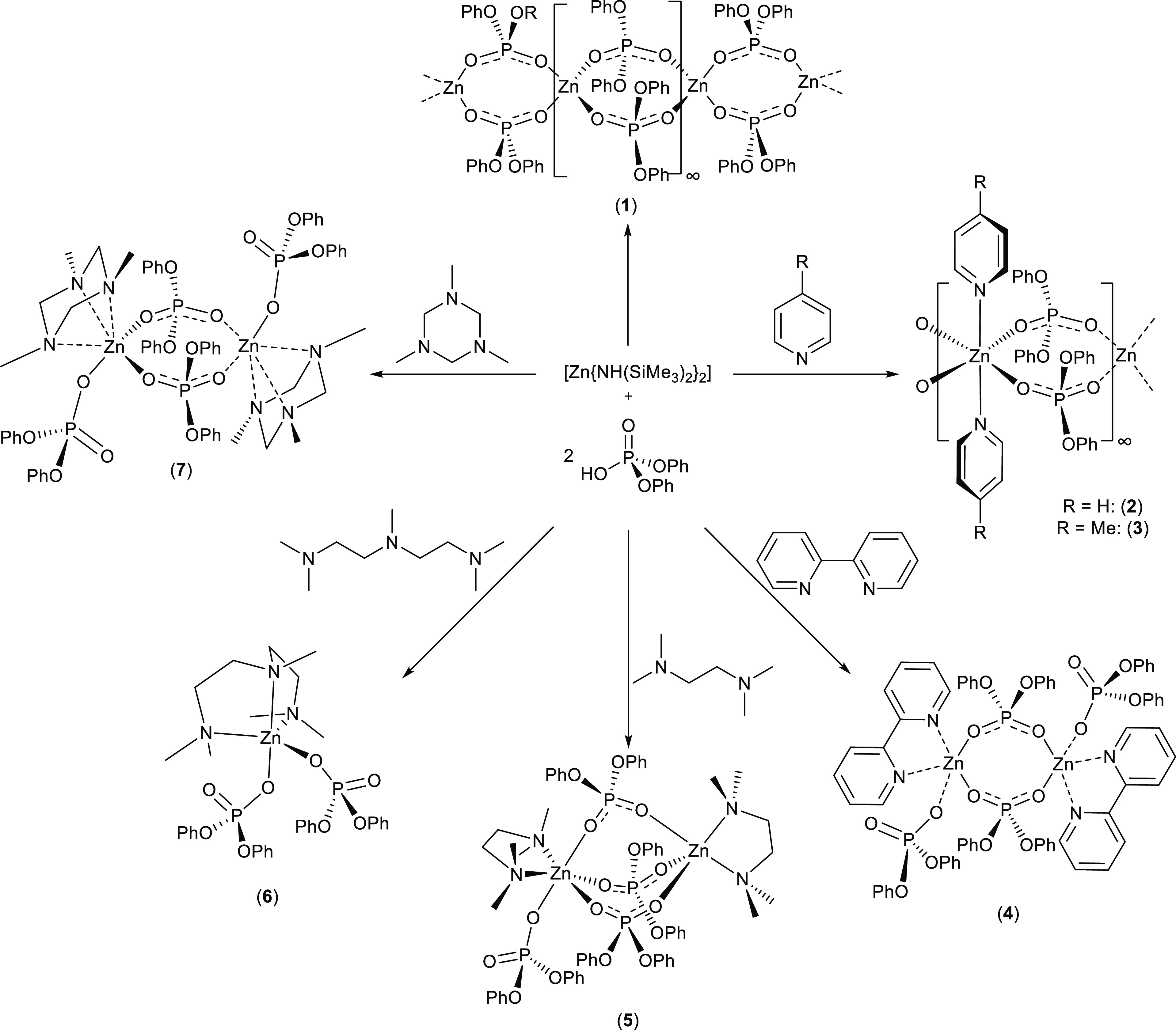
Synthesis and Solid-State Molecular Structures of Zinc Phosphate Complexes **1**–**7**

Herein, we report the preliminary experiments involving the reactions of zinc bis(hexamethyldisilazide), [Zn(N{SiMe_3_}_2_)_2_], with the organophosphate diester HO(O)P(OPh)_2_. In an attempt to gain insights into the factors determining reaction outcomes and the molecular structure of the resulting zinc phosphates, we have explored the role of co-ligands in zinc phosphate complex formation, choosing a series of Lewis basic nitrogen ligands of varying denticity: pyridine (Py), 4-methylpyridine (4-MePy), 2,2-bipyridine (bipy), tetramethylethylenediamine (TMEDA), pentamethyldiethylenetriamine (PMDETA), and 1,3,5-trimethyl-1,3,5-triazacyclohexane (Me_3_-TAC) (Scheme 1). To increase our understanding of these systems, SCXRD has been compared to data from ^1^H DOSY NMR experiments, used to determine the molecular weight of the species in solution for complexes **3–7**, thereby identifying the aggregation states and relative distribution of the Lewis basic nitrogen ligands, zinc centers, and diphenyl phosphate anions in the solution state.

## Results and Discussion

### Synthesis and Solid-State Structures

**Scheme 2:**
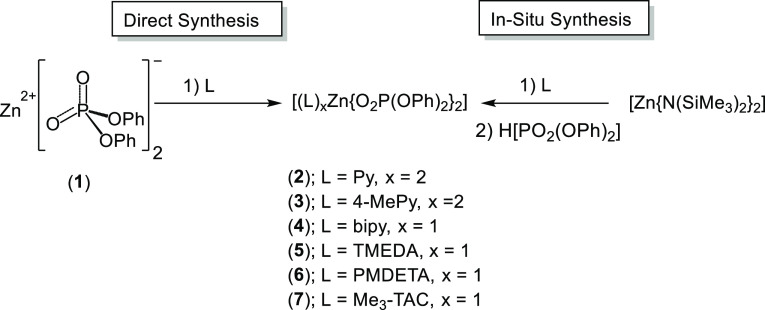
Direct and *In Situ* Synthetic Methodologies Used in the Synthesis of Complexes **1–7**

All complexes were initially synthesized using an *in situ* “acid–base” synthetic route, in which the base, [Zn(N{SiMe_3_}_2_)_2_], reacted with the acid, HO(O)P(OPh)_2_, in the presence of a ligand. Direct methods of synthesis, whereby the pre-formed salt, [Zn{O_2_P(OPh)_2_}_2_] (**1**), is treated with the Lewis base (co-ligand) either neat or in THF solution, can also be applied to these systems (Scheme 2).

Both direct and *in situ* methods of zinc complex preparation were investigated to reveal any differences in products formed by the two methods, *e.g.*, different stoichiometries, alternative coordination modes of co-ligands, and polymorphism, *etc.* Our investigation has shown that for the compounds discussed here, both methodologies are equally effective, and for comparable experiments, products were found to be identical by multinuclear NMR spectroscopy, elemental analysis, and unit cell determination. ^1^H, ^13^C, and ^31^P NMR solution-state experiments performed on complexes **1**–**7** indicate that in all cases, on the chemical shift time scale, the zinc center appears to remain coordinated to the nitrogen co-ligand. As for the phosphate ligands, NMR spectra (^31^P, ^1^H, and ^13^C) provide little information about the coordination mode of the [O_2_P(OPh)_2_]^−^ anion in the solution state.

**Figure 2:**
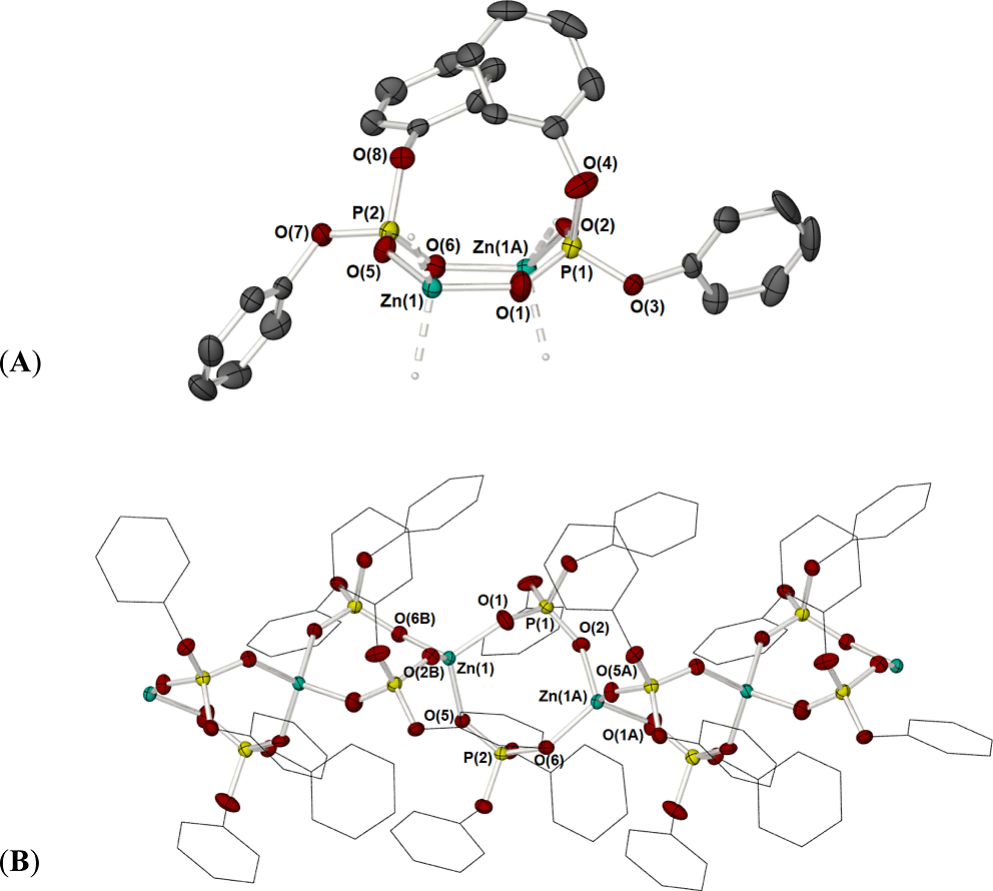
(A) Molecular structure of (1) highlighting the “boat-like” conformation of the eight-membered {Zn_2_{OPO}_2_} ring. (B) Partially labeled view of the 1D polymeric chain of [Zn{O_2_P(OPh)_2_}_2_]_∞_ (**1**). Hydrogen atoms have been omitted, and phenyl groups have been shown as wire frames for clarity. Thermal ellipsoids are shown at 50% probability. Equivalent atoms (A and B) are generated by the symmetry operators A: −*x* + *y*, 1 – *x*, 1/3 + *z* and B: 1 – *y*, 1 + *x* + *y*, *z* – 1/3.

The zinc bisphosphate salt, **1**, is readily formed by the stoichiometric reaction of [Zn(N{SiMe_3_}_2_)_2_] with two equivalents of HO(O)P(OPh)_2_ in THF at 0 °C, leading to the formation of a clear colorless solution. Removal of the solvent under vacuum followed by dissolution in a minimum of hot toluene and hot filtration yielded a colorless solution from which colorless crystals formed upon standing at 4 °C. Needle-like crystals suitable for X-ray diffraction analysis were isolated from the reaction mixture. SCXRD experiments reveal the asymmetric unit cell of **1**. Figure 2A consists of a single zinc atom coordinated by two oxygen atoms, one from each of the two {O_2_P(OPh)_2_} units. Crystallographic symmetry renders the asymmetric unit part of an infinite 1D helical chain of {ZnO_4_} and {PO_4_} tetrahedra, as expressed by the trigonal space group *P*3_2_, in which complex **1** crystalizes. A partially labeled view of a section of the polymer chain is shown in Figure 2B. Relevant bond lengths and bond angles are presented in Table 1. The gross structural features of **1** are comparable to those of the related complexes, [Zn{O_2_P(OMe)_2_}_2_]_∞_^17b^ and [Zn{O_2_P(OEt)_2_}_2_]_∞_,^17c^ where each, approximately orthogonal, eight-membered heterocyclic {Zn_2_(OPO)_2_} ring is linked together at the zinc centers, to form a spirocyclic coordination polymer.

**Table 1:** Selected Bond Lengths (Å) and Angles (°) for Complexes **1**

Bond Lengths (Å)			
Zn(1)–O(1)	1.922(6)	P(1)–O(1)	1.474(6)
Zn(1)–O(5)	1.930(4)	P(1)–O(2)	1.493(5)
Zn(1)–O(2A)^a^	1.938(4)	P(1)–O(3)	1.569(4)
Zn(1)–O(6A)^a^	1.940(5)	P(1)–O(4)	1.591(5)
		P(2)–O(5)	1.475(5)
Zn(1)···Zn(1A)^a^	4.383(10)	P(2)–O(6)	1.495(5)
		P(2)–O(7)	1.582(4)
		P(2)–O(8)	1.587(5)
Bond Angles (deg)			
O(1)–Zn(1)–O(2A)^a^	103.1(2)	O(2)–P(1)–O(3)	111.5(4)
O(1)–Zn(1)–O(6B)^b^	113.1(2)	O(2)–P(1)–O(4)	108.6(3)
O(1)–Zn(1)–O(5)	109.8(2)	O(3)–P(1)–O(4)	101.8(3)
O(5)–Zn(1)–O(2A)^a^	114.8(2)		
O(5)–Zn(1)–O(6A)^a^	104.4(2)	O(5)–P(2)–O(6)	117.3(3)
		O(5)–P(2)–O(7)	109.7(3)
O(1)–P(1)–O(2)	116.8(3)	O(5)–P(2)–O(8)	107.1(3)
O(1)–P(1)–O(3)	104.8(3)	O(6)–P(2)–O(7)	109.5(3)
O(1)–P(1)–O(4)	111.5(4)	O(6)–P(2)–O(8)	111.2(3)
		O(7)–P(2)–O(8)	100.8(2)

a Equivalent atoms are generated by the symmetry operators -x+y, 1-x, 1/3+z.

b Equivalent atoms are generated by the symmetry operators 1-y, 1+x+y, z-1/3.

Each zinc atom is tetrahedrally coordinated: *d*[Zn(1)–O(1)] = 1.922(6) Å, *d*[Zn(1)–O(2)$] = 1.930(4) Å, *d*[Zn(1)–O(5)] = 1.938(4) Å, *d*[Zn(1)–O(6)$] = 1.940(5) Å. The coordination sphere about each zinc atom is completed by oxygen atoms from the adjacent unit cell, such that the phosphate ligands bridge zinc centers in a {μ-O,O′} fashion, forming a continuous series of eight-membered {Zn_2_(OPO)_2_} rings, with a boat-like conformation (Figure 2) which propagates along the crystallographic *c*-axis. The phosphorus centers show typical {PO_4_}, tetrahedral geometry: for each P atom, two P–O bonds connect to adjacent zinc atoms, and the other two P–O vertices link to “terminal” {Ph} groups. A characteristic difference in the bond length is apparent between these two P–O chemical environments (Table 1).

**Figure 3:**
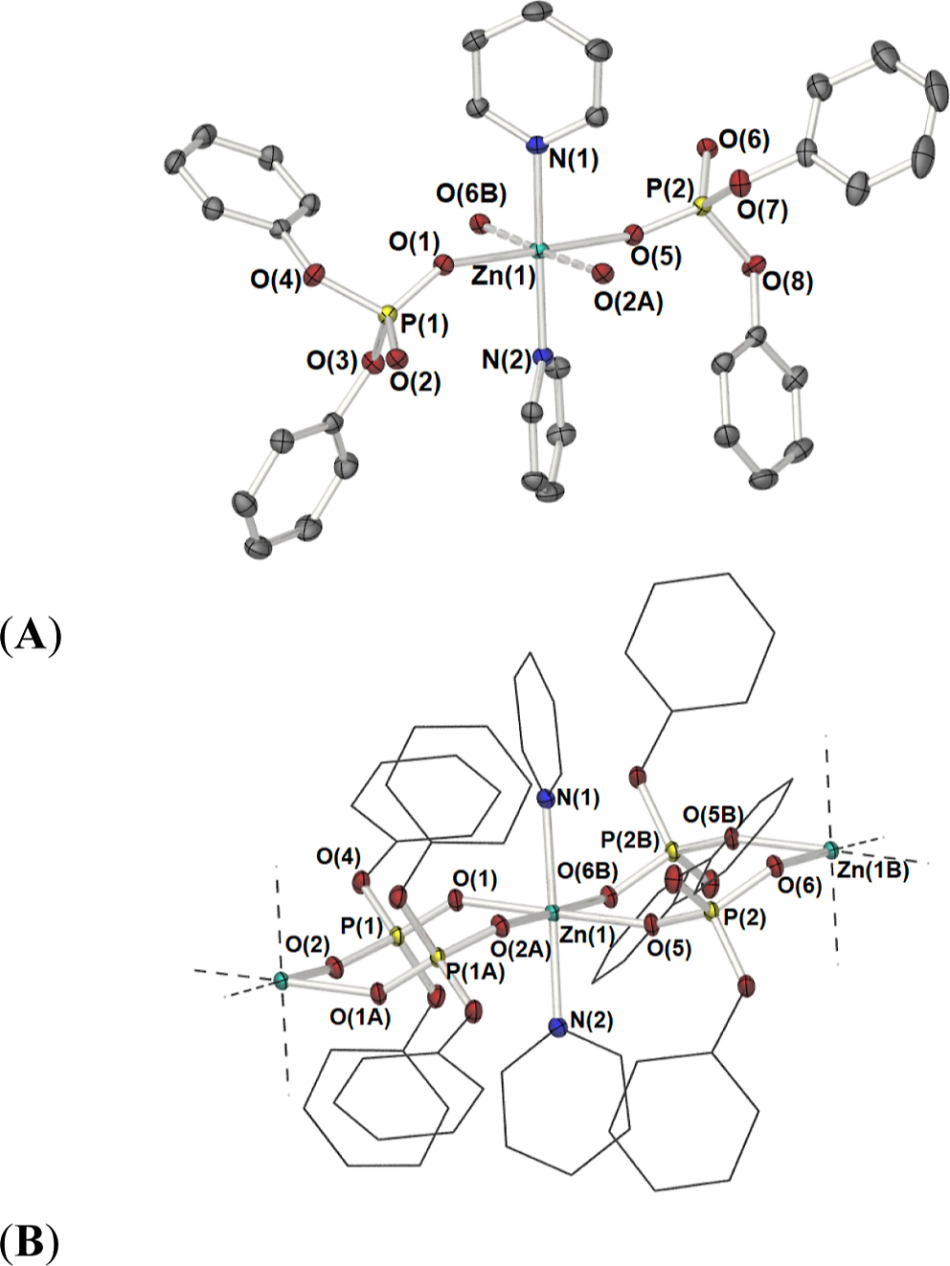
Partially labeled plot of the asymmetric unit cell contents of **2**, [Zn(Py)_2_{μ-O_2_P(OPh)_2_}_2_]_∞_ (A), and a view of the polymeric 1D chain parallel to the *a*-axis. (B) {Ph} and {Py} moieties are shown as the wire framework, for clarity. Equivalent atoms are generated by the symmetry operators A: 1 – *x*, 1 – *y*, 1 – *z* and B: 2 – *x*, 1 – *y*, 1 – *z*.

Crystals of both the pyridine and methylpyridine adducts of [Zn{O_2_P(OPh)_2_}], **2** and **3** respectively, were grown from the reaction mixtures at 4 °C. Despite complexes **2** and **3** crystallizing in different crystallographic space groups, they possess very similar structural motifs. Complex **2**, as shown in Figure 3, with selected bond lengths and angles listed in Table 2, crystalizes in the triclinic *P*1̅ space group, with structural analysis revealing the formation of a 1D polymeric Zn(II) complex, the asymmetric unit cell of which contains one formula unit per unit cell, *i.e.*, [Zn(*trans*-Me-Py)_2_{*trans*-μ-κ^2^-O_2_P(OPh)_2_}_2_] alongside one molecule of uncoordinated THF. The cores of **2** and **3**, consist of {*trans*-Zn(Py)_2_} [Py = C_5_H_5_N (**2**) or 4-Me-C_5_H_4_N (**3**)] moieties supported by transoidal bridging diphenyl phosphate ligands {O_2_P(OPh)_2_}, thus forming an infinite chain of eight-membered {Zn_2_(OPO)_2_} rings, with chair-like conformations (Figure 3) which propagate along the crystallographic *a*-axis (Figure 3a). In both **2** and **3**, the octahedral geometry about each Zn(II) center is completed by coordination through oxygen atoms of the phosphate ligand to an adjacent zinc center. [For Zn(*trans*-Py)_2_{*trans*-μ-κ^2^-O_2_P(OPh)_2_}_2_] (**2**); *d*[Zn(1)–N(1)] = 2.1400(14) Å, *d*[Zn(1)–N(2)] = 2.1365(14) Å, *d*[Zn(1)–O(1)] = 2.0941(12) Å, *d*[Zn(1)–O(2A)] = 2.1326(11) Å, *d*[Zn(2)–O(5)] = 2.1014(12) Å, *d*[Zn(1)–O(6A)] = 2.1100(11) Å; ∠[N(1)–Zn(1)–N(2)] = 178.30(5)°, ∠[O(1)–Zn(1)–O(5)] = 178.31(4)°, ∠[O(6A)–Zn(1)–O(2B)] = 177.04(5)°. The molecular structure of the asymmetric unit cell and a fragment of the 1D polymeric chain are shown in Figure 3.

**Table 2:** Selected Bond Lengths (Å) and Angles (°) for Complex **2**^a^

Bond Lengths (Å)				
Zn(1)–N(1)	2.1400(14)	P(1)–O(1)	1.4782(12)	
Zn(1)–N(2)	2.1365(14)	P(1)–O(2)	1.4826(12)	
Zn(1)–O(1)	2.0941(12)	P(1)–O(3)	1.6112(12)	
Zn(1)–O(5)	2.1014(12)	P(1)–O(4)	1.6049(12)	
Zn(1)–O(2A)^a^	2.1326(11)	P(2)–O(5)	1.4702(13)	
Zn(1)–O(6B^a^)	2.1100(11)	P(2)–O(6)	1.4859(12)	
		P(2)–O(7)	1.6100(12)	
Zn(1)···Zn(1B)	5.209(3)	P(2)–O(8)	1.6080(13)	
Bond Angles (°)				
N(1)–Zn(1)–N(2)	178.30(5)	O(1)–P(1)–O(2)	119.80(7)	
O(1)–Zn(1)–O(5)	178.31(4)	O(3)–P(1)–O(4)	105.38(7)	
O(2A)–Zn(1)–O(6B)^a^	177.04(5)	O(5)–P(2)–O(6)	121.03(7)	
		O(7)–P(2)–O(8)	102.36(7)	

a Equivalent atoms (A and B) are generated by the symmetry operators: (A) 1 – *x*, 1 – *y*, 1 – *z* and (B) 2 – *x*, 1 – *y*, 1 – *z*.

In contrast to **2**, complex **3** crystalizes in the monoclinic space group *P*2_1_/*c* with two formula units in the unit cell, *i.e.*, [Zn(*trans*-Me-Py)_2_{*trans*-μ-k^2^-O_2_P(OPh)_2_}_2_]_2_ and no solvent of crystallization. As with **2**, interaction with adjacent zinc centers results in the formation of an infinite chain of eight-membered {Zn_2_O_4_P_2_} rings, with a chair-like conformation, linked by {*trans*-(Me-Py)_2_Zn} units. The molecular structure of **3** along with selected bond lengths and angles is shown in the Supporting Information. In both **2** and **3**, the pyridine rings coordinated to adjacent Zn(II) centers are involved in a weak bifurcated edge-to-face (C–H/π: 3.133–3.751 Å) interactions between adjacent pyridine rings.^21^

**Figure 4:**
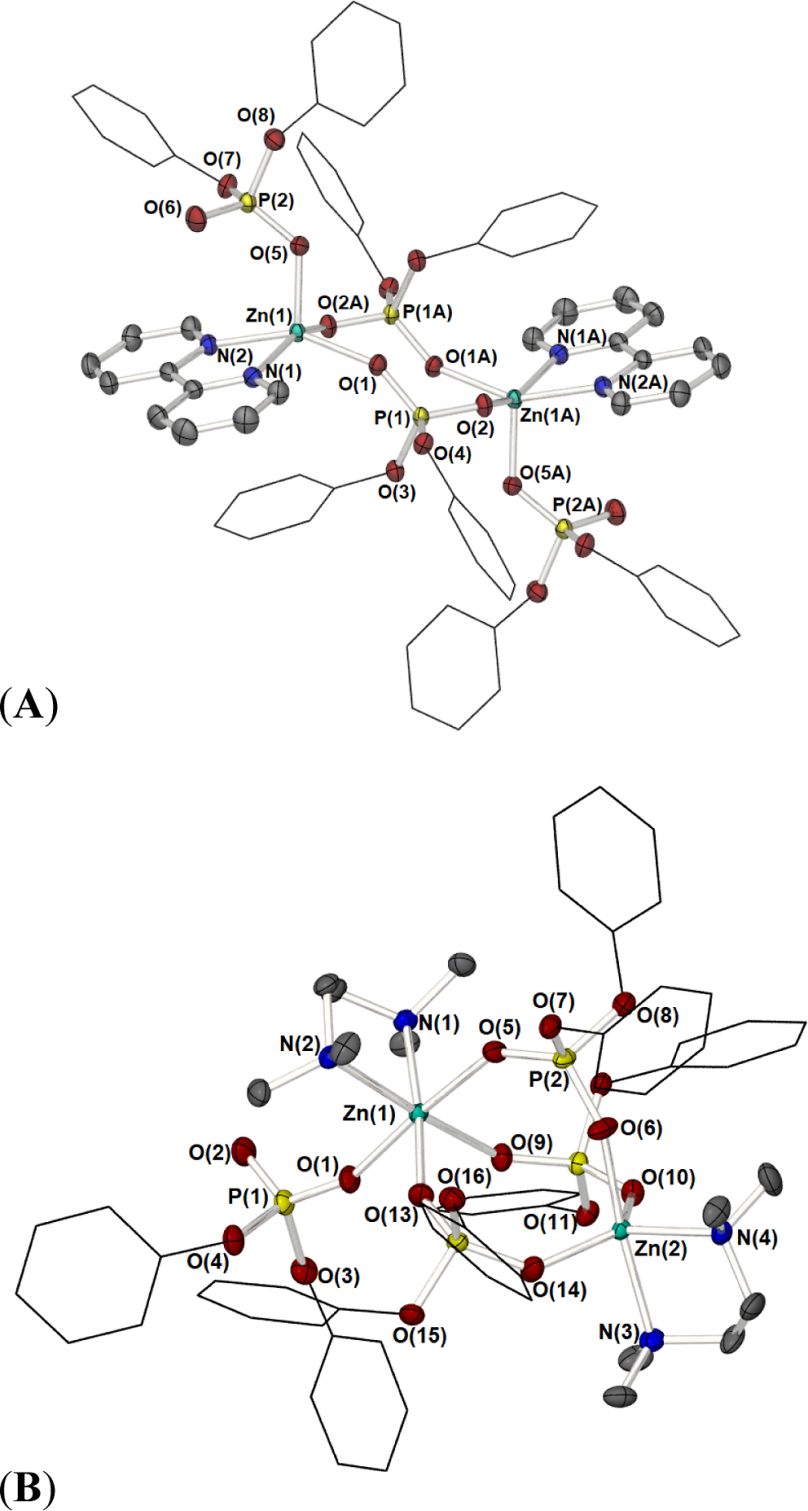
Partially labeled molecular structure plots of complexes **4** (A) and **5** (B). Thermal ellipsoids are shown at 50% probability. Hydrogen atoms have been omitted, and the {Ph} groups are shown as wire frameworks for clarity. Equivalent atoms in **4** are generated by the symmetry operator A: 1 – *x*, 1 – *y*, 1 – *z*.

On changing the nitrogen donor ligand from monodentate (Py or Me-Py) to the bidentate ligands 2,2′-Bipy and TMEDA, separately, the aggregation state of the zinc phosphate is reduced, with the formation of the bimetallic complexes [(Bipy)·Zn(m-OP(OPh)_2_O)]_2_ (**4**) and [(TMEDA)_2_·Zn_2_(μ-OP(OPh)_2_O)_3_(k^1^-OP(O)(OPh)_2_)] (**5**), respectively. The solid-state molecular structures of complexes **4** and **5** are shown in Figure 4, with selected bond lengths and angles provided in Table 3. Single crystals for the X-ray diffraction of **4** were obtained by crystallization of the sample from a layered DCM/hexane solution at room temperature, the compound crystallizing in the triclinic space group *P*1̅. The asymmetric unit cell contains two independent subunits, [(Bipy)·Zn{O_2_P(OPh)_2_}_2_], related by centers of inversion. The bimetallic nature of the complex is reminiscent of the phenanthroline adduct reported by Murugavel et al.,^16d^ where two zinc atoms are linked to each other by two di-phenyl phosphate ligands, as shown in Figure 4a. The zinc atoms are all five-coordinate, with a square-pyramidal geometry (structural index of τ = 0.03)^22^ where the apical position is occupied by an oxygen atom of a terminal diphenyl phosphate ligand *d*[Zn–O(5) 1.9648(10) Å]. The basal plane of the zinc coordination sphere consists of the two nitrogen atoms of the 2,2′-bipyridyl ligand and two bridging di-phenyl phosphate oxygen atoms. See Table 3 for selected bond lengths and bond angles.

**Table 3:** Selected Bond Lengths (Å) and Angles (°) for Complexes **4** and **5**

Bond Lengths (Å)		Bond Angles (deg)		
**4**				
Zn(1)–N(1)	2.1155(12)	N(1)–Zn(1)–N(2)	77.28(5)	
Zn(1)–N(2)	2.1090(13)	N(1)–Zn(1)–O(1)	89.58(4)	
Zn(2)–N(3)	2.1384(12)	N(1)–Zn(1)–O(2A)	153.20(5)	
Zn(2)–N(4)	2.1022(12)	N(1)–Zn(1)–O(5)	105.67(5)	
Zn(1)–O(1)	2.0219(10)	N(2)–Zn(1)–O(2A)	88.58(4)	
Zn(1)–O(2A)^a^	2.0348(10)	N(2)–Zn(1)–O(1)	151.58(5)	
Zn(1)–O(5)	1.9648(10)	O(1)–Zn(1)–O(2A)	92.54(4)	
O(1)–P(1)	1.4855(10)	N(1)–Zn(1)–O(5)	105.67(5)	
O(2)–P(1)	1.4879(10)	N(2)–Zn(1)–O(5)	103.85(5)	
O(3)–P(1)	1.5927(11)	O(1)–Zn(1)–O(5)	103.93(4)	
O(4)–P(1)	1.5990(10)	O(2A)–Zn(1)–O(5)	99.73(4)	
O(5)–P(2)	1.4972(11)	O(1)–P(1)–O(2)	118.55(6)	
O(6)–P(2)	1.4667(11)	O(3)–P(1)–O(4)	105.32(6)	
O(7)–P(2)	1.6105(10)	O(5)–P(2)–O(6)	120.35(7)	
O(8)–P(2)	1.6048(11)	O(7)–P(2)–O(8)	103.99(6)	
Zn(1)···Zn(1A)^a^	5.31220(11)			
**5**				
Zn(1)–N(1)	2.2289(12)	N(1)–Zn(1)–N(2)	82.49(5)	
Zn(1)–N(2)	2.2199(12)	O(9)–Zn(1)–O(13)	93.64(4)	
Zn(1)–O(1)	2.0824(10)	N(1)–Zn(1)–O(13)	173.92(5)	
Zn(1)–O(5)	2.1590(10)	N(2)–Zn(1)–O(9)	174.79(5)	
Zn(1)–O(9)	2.0625(10)	O(1)–Zn(1)–O(5)	175.82(4)	
Zn(1)–O(13)	2.0673(10)	N(3)–Zn(2)–N(4)	83.36(5)	
Zn(2)–N(3)	2.2056(13)	O(10)–Zn(2)–O(14)	127.76(5)	
Zn(2)–N(4)	2.1092(12)	O(6)–Zn(2)–N(3)	173.10(5)	
Zn(2)–O(6)	2.0353(11)	N(4)–Zn(2)–O(10)	111.26(5)	
Zn(2)–O(10)	1.9879(10)	N(4)–Zn(2)–O(14)	120.73(5)	
Zn(2)–O(14)	1.9840(11)	O(1)–P(1)–O(2)	121.21(7)	
O(1)–P(1)	1.4820(10)	O(3)–P(1)–O(4)	101.67(6)	
O(2)–P(2)	1.4675(12)	O(5)–P(2)–O(6)	121.82(6)	
O(5)–P(2)	1.4765(11)	O(7)–P(2)–O(8)	103.68(6)	
O(6)–P(2)	1.4815(11)	O(9)–P(3)–O(10)	119.85(6)	
O(9)–P(3)	1.4739(10)	O(11)–P(3)–O(12)	103.58(6)	
O(10)–P(3)	1.4912(10)	O(13)–P(4)–O(14)	120.25(6)	
O(13)–P(4)	1.4707(11)	O(15)–P(4)–O(16)	103.02(6)	
O(14)–P(4)	1.4914(11)			
Zn(1)···Zn(2)	4.500049(3)			

a Equivalent atoms in 4 are generated by the symmetry operator A: 1-x, 1-y, 1-z

Compound **5** crystalizes from the reaction mixture at −28 °C, in the monoclinic space group *P*2_1_/*c* with one whole dimer molecule in the asymmetric unit cell, and possesses a {Zn(m-OP(OPh)_2_O)_3_Zn} core reminiscent of [Zn_2_{m-O_2_P(O^t^Bu)}_3_(OP(OtBu)_2_O)]_*n*_ (Figure 1b), in which three phosphate {O_2_P(OPh)_2_} ligands bridge the two zinc centers, with the fourth diphenyl phosphate ligand occupying a terminal position about one of the two zinc atoms. The solid-state molecular structure of **5** is shown in Figure 4b. A κ^2^-*N*,*N* coordination of a TMEDA ligand to each zinc atom completes the coordination sphere of each zinc atom.

As a result, one zinc atom [Zn(1)] is octahedrally coordinated, with the nitrogen atoms of the TMEDA ligand occupying two cisoidal positions about the zinc atom. The second zinc atom possesses a pseudo-trigonal bipyramidal 5-coordinate environment (τ = 0.76),^22^ with the two oxygen atoms of the bridging phosphate groups occupying equatorial positions along with one nitrogen atom of a TMEDA ligand *d*[Zn(2)–N(4) 2.1092(12) Å]. The longer axial positions about Zn(2) are occupied by the second nitrogen atom of the TMEDA ligand and the third oxygen atom of a bridging {O_2_P(OPh)_2_} ligand. See Table 3 for the selected bond lengths and bond angles.

**Figure 5:**
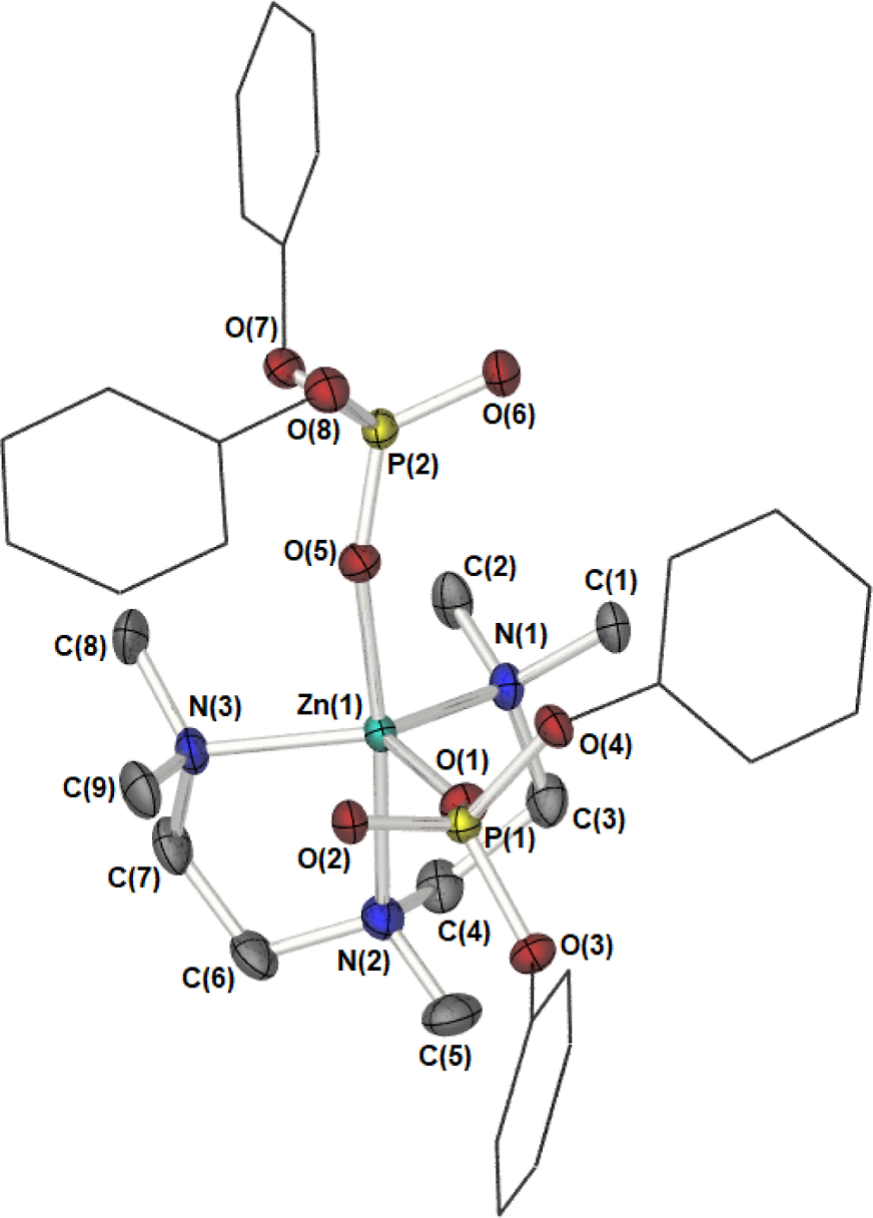
Partially labeled molecular structure plot of the monomeric species [(PMDETA)·Zn{O_2_P(OPh)_2_}_2_], **6**. Thermal ellipsoids are shown at 50% probability. Hydrogen atoms have been omitted, and phenyl groups are shown as wire frameworks for clarity.

On changing the nitrogen donor ligand from bidentate TMEDA to the tridentate ligand PMDETA, the aggregation state of the zinc bis-diphenylphosphate is further reduced. Single-crystal X-ray analysis of crystals grown from the reaction mixture at room temperature reveals a monomeric complex [(PMDETA)·Zn{O_2_P(OPh)_2_}_2_] (**6**), the solid-state molecular structure of which is shown in Figure 5. Selected bond lengths and angles for **6** are shown in Table 4.

**Table 4:** Selected Bond Lengths (Å) and Angles (°) for Complex **6**

Bond Lengths (Å)			
Zn(1)–N(1)	2.0668(12)	P(1)–O(1)	1.4911(11)
Zn(1)–N(2)	2.2806(13)	P(1)–O(2)	1.4648(11)
Zn(1)–N(3)	2.1020(13)	P(1)–O(3)	1.6124(11)
Zn(1)–O(1)	1.9430(11)	P(1)–O(4)	1.6089(11)
Zn(1)–O(5)	2.0285(11)	P(2)–O(5)	1.4851(11)
		P(2)–O(6)	1.4672(11)
		P(2)–O(7)	1.6189(11)
		P(2)–O(8)	1.6141(11)
Bond Angles (deg)			
O(1)–Zn(1)–N(1)	109.27(5)	O(1)–P(1)–O(2)	122.38(7)
O(1)–Zn(1)–N(3)	124.19(5)	O(3)–P(1)–O(4)	104.48(6)
N(1)–Zn(1)–N(3)	123.78(5)		
O(1)–Zn(1)–O(5)	97.79(5)	O(5)–P(2)–O(6)	122.32(7)
O(5)–Zn(1)–N(2)	172.36(5)	O(7)–P(2)–O(8)	103.41(6)

The asymmetric unit cell, (*P*2_1_/*c*), comprises one whole molecule in which the central zinc cation possesses a five-coordinate pseudo-trigonal bipyramidal geometry (τ = 0.81),^22^ with the PMDETA ligand bound to the zinc center in a tridentate facial-coordination mode with Zn–N bond lengths similar to previous reported values.^23^ The remaining axial and equatorial positions about the zinc center are occupied by two independent oxygen atoms of the two terminal phosphate ligands *d*[Zn(1)–O(1) 1.943(10) Å and Zn(1)–O(5) 2.028(10) Å]. See Table 4 for selected bond lengths and bond angles.

Distortion away from an ideal trigonal bipyramidal geometry in **6** is manifested in the axial-equatorial bond angles and is presumably the result of constrained bite angles within the PMDETA ligand and the steric bulk of the diphenylphosphate anions (see Table 4). Finally, we wished to investigate the effect of variation in the nature of the tridentate donor ligand by synthesizing complexes of *N*,*N*′,*N*″-trimethyltriazocyclohexane (Me_3_-TAC). Although both PMDETA and Me_3_-TAC contain three donor atoms, we expected the more compact ligand set of Me_3_-TAC, relative to PMDETA, to have a significant influence over the nature of aggregation in weakly coordinating salt adducts, as has been observed previously.^24^ To date, only a very small number of structurally characterized Zn-TAC complexes are known in the literature and include both the sandwich-like bis-TAC complexes of the form [(TAC)_2_Zn]^2+^ and “half-sandwich” [(TAC)-Zn]^2+^ systems.

**Figure 6:**
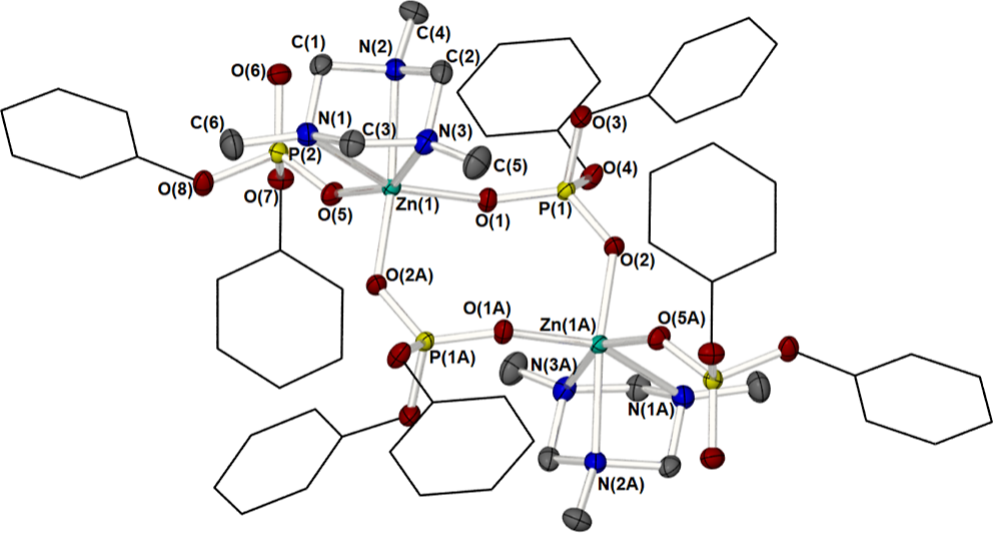
Partially labeled molecular structure plot of the dimeric species [{Me_3_-TAC}·Zn{O_2_P(OPh)_2_}_2_]_2_, **7**. Thermal ellipsoids are shown at 50% probability. Hydrogen atoms have been omitted, and phenyl groups are shown as wire frameworks for clarity. Equivalent atoms in **7** are generated by the symmetry operator A: −*x*, 1 – *y*, 1 – *z*.

Complex **7** was characterized by SCXRD, the solid-state molecular structure of which is shown in Figure 6 and selected bond lengths and angles are provided in Table 5.

**Table 5:** Selected Bond Lengths (Å) and Angles (°) for Complex **7**

Bond Lengths (Å)		Bond Angles (°)	
Zn(1)–N(1)	2.3512(12)	N(1)–Zn(1)–N(2)	60.71(4)
Zn(1)–N(2)	2.2396(12)	N(1)–Zn(1)–N(3)	59.23(4)
Zn(1)–N(3)	2.3802(13)	N(2)–Zn(1)–N(3)	60.10(4)
Zn(1)–O(1)	2.0013(10)	O(1)–Zn(1)–O(2A)	99.99(4)
Zn(1)–O(2A)^a^	1.9908(10)	O(1)–Zn(1)–O(5)	98.97(4)
Zn(1)–O(5)	1.9824(10)	O(2A)–Zn(1)–O(5)	99.63(4)
		O(1)–Zn(1)–N(1)	155.43(4)
Zn(1)···Zn(1A)^a^	4.89862(4)	O(2A)–Zn(1)–N(2)	148.78(5)
		O(5)–Zn(1)–N(3)	155.70(4)
O(1)–P(1)	1.4800(10)		
O(2)–P(1)	1.4864(9)		
O(3)–P(1)	1.5948(11)	O(1)–P(1)–O(2)	120.61(6)
O(4)–P(1)	1.5983(10)	O(3)–P(1)–O(4)	104.15(6)
O(5)–P(2)	1.4893(10)		
O(6)–P(2)	1.4699(10)	O(5)–P(2)–O(6)	121.76(6)
O(7)–P(2)	1.6095(10)	O(7)–P(2)–O(8)	103.30(6)
O(8)–P(2)	1.6166(10)		

a Equivalent atoms in 7 are generated by the symmetry operator A: -x, 1-y, 1-z.

The monoclinic asymmetric unit cell (*P*2_1_/*c*) contains one half of a phosphate bridged dimer, [η^3^-{Me_3_-TAC}·Zn{m-OP(OPh)_2_O}{O_2_P(OPh)_2_}]_2_ (**7**), such that two Zn centers are linked by bridging diphenylphosphate ligands thus forming an eight-membered {Zn_2_(OPO)_2_} core. The dimeric nature of the complex is comparable to both the {Phen}^16d^ and the {bipy} complex, **4**, reported here, where two zinc atoms are linked to each other by two di-phenyl phosphate ligands, as shown in Figure 6. However, in the case of **7**, the zinc atoms are both six-coordinate, with a distorted octahedral geometry. Here, three of the octahedral sites about the Zn atom are occupied by the nitrogen atoms of the (TAC) ligand. See Table 5 for selected bond lengths and bond angles. The Zn–N bond lengths are longer than those reported in either **4** or **6**, suggesting a weaker coordination, an observation reflected in the shorter Zn(1)–O(1) bond lengths. There are now, for zinc, several TAC adducts with which the coordination geometry at zinc in the present compound can be compared.^25^

A consequence of complexation of the (TAC) ligand to the Zn center is a reorientation of the nitrogen lone pairs toward the metal, such that the *N*-methyl groups are lowered in the direction of the zinc center. Köhn et al. previously noted that this bending can be quantified by the value Δ, a measure of orientation, or bond bending, of the lone pair of electrons on the nitrogen atom toward the metal center.^25b,26^ The free ligand has a Δ value of 0.482 Å (a perfect tetrahedron has an expected value of Δ = 0.49 Å), while **7** shows Δ values ≈ 0.22 Å indicating significant bending.^27^ Analysis also shows that the constrained bite angle of the ligand results in a significant distortion away from octahedral geometry, most obviously reflected in the *trans*-N–Zn–O bond angles [Ave N–Zn–N ∼ 153°]. Irrespective of reaction stoichiometries, there was no evidence of the formation of a charge-separated species of the form [{Me_3_-TAC}_2_Zn][O_2_P(OPh)_2_]_2_.

### Solution Phase Characterization

As noted above, in an attempt to elucidate the solution phase structures of complexes **1**–**7** and to deduce whether the complexation observed in the solid state persists in solution, a series of NMR studies were performed. Analysis of 1D NMR data demonstrates that such systems show fluxional dynamic behavior in solution. For example, the solid-state structure of complex **4** displays multiple phosphate ligand environments, yet ^31^P{^1^H} NMR studies in CD_2_Cl_2_ reveal only one resonance in the spectra, indicating probable exchange in solution on the ^31^P{^1^H} NMR time scale. Similar observations are made for complex **3** and **5**–**7**.

In an attempt to determine the oligomeric nature of the zinc-diphenyl phosphate Lewis base adducts in solution, ^1^H DOSY experiments were completed and analyzed using the ECC-MW methodology described by Stalke et al.^4b^

Here, the application of a merged compact spheres/dissipated spheres and ellipsoids/extended discs calibration curve was best suited to non-polarizable Zn^2+^ containing contact ion pair systems and provided the best fit to the selected systems shown in Table 6. It should be noted that DOSY NMR experiments have previously been used to elucidate the solution-state structures and degree of aggregation in a series of ethyl-zinc pyrazole derivatives.^28^

**Table 6:** Normalized Diffusion Coefficients (*D*_norm_), Calculated Molecular Weights (MW_Cal_), Molecular Weights Determined by ^1^H DOSY [MW_det_ (^1^H)] and Error in MW between the Calculated and Observed Values (MW_err_) for Reference Compounds Used in This Study at 298 K

Compound	NMR Solvent	Observed Diffusion Coefficient (m^2^ s^–1^)	MW_Cal_ (g mol^–1^)	MW_det_ (g mol^–1^)	MW_err_^a^ (%)
[HO(O)P(OPh)_2_]_2_	C_6_D_6_	7.07 × 10^–10^	500	511	2
[Zn(S_2_CNEt_2_)_2_]	C_6_D_6_	9.34 × 10^–10^	362	314	15
[Zn({OC_6_H_2_^*t*^Bu_2_CH_2_}_2_NC_2_H_4_NMe_2_)]	C_6_D_6_	6.40 × 10^–10^	588	594	1
Zn(TMHD)2	(CD_3_)_2_SO	9.34 × 10^–10^	432	503	14

a MW_err_ = [100 × (MW_cal_ – MW_det_)/MW_cal_].

**Figure 7:**
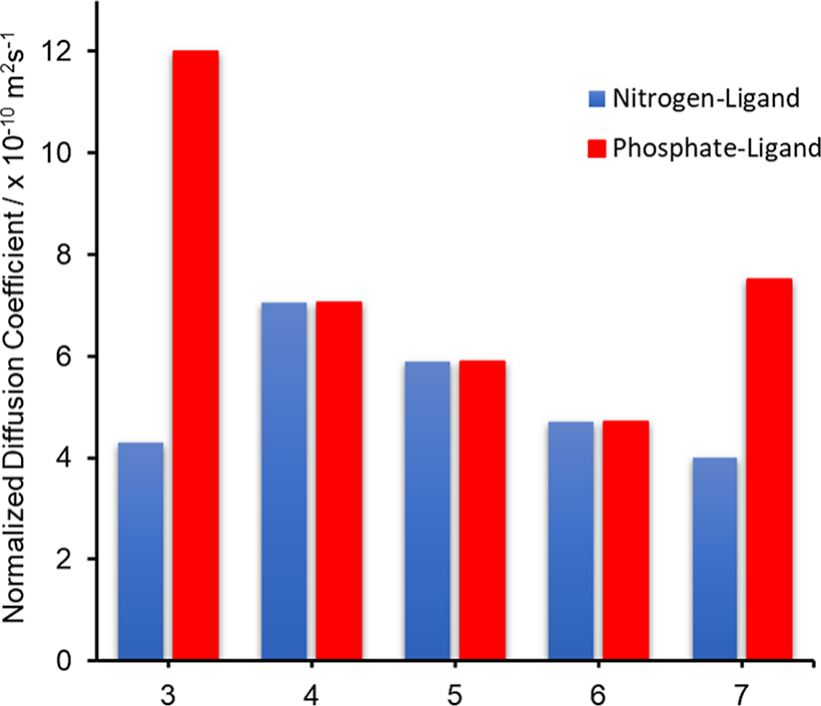
Plot showing the normalized diffusion coefficients for nitrogen and phosphate ligand containing species in solutions of compounds **3**, **4**, **5**, **6**, and **7**, as determined by ^1^H DOSY NMR at 298 K.

Compounds **3–7** were subsequently interrogated using ^1^H DOSY NMR spectroscopy, using a merged calibration curve to estimate the molecular weight of species present in solution. Unfortunately, compounds **1** and **2** were found to be insufficiently soluble in either C_6_D_6_, *d*_8_-tol, CDCl_3_ or CD_2_Cl_2_ to produce meaningful data. Figure 7 shows the diffusion coefficients, as calculated from ^1^H NMR DOSY experiments for complexes **3**, **4**, **5**, **6**, and **7** (Table 7), at 298 K, in either C_6_H_6_ (**3**, **5**, **6** and **7**) or CD_2_Cl_2_ for (**4**). In the case of complex **4**, insufficient solubility in C_6_D_6_ prompted the use of CD_2_Cl_2_. All spectra and diffusion data are included in the Supporting Information (Figures S2–S6 and Tables S3–S7).

**Table 7:** Normalized Diffusion Coefficients (*D*_norm_), Calculated Molecular Weights (MW_Cal_), Molecular Weights Determined (by ^1^H DOSY) [MW_det_ (^1^H)], Proposed Solution State Structures Based on MW, and Errors in MW between the Calculated and Observed Values (MW_err_) for **3**–**7** at 298 K

Compound	Nitrogen-donor ligand	NMR Solvent	Normalized Diffusion Coefficient (*D*_norm_) (m^2^ s^–1^)	MW_det_ (^1^H) (g mol^–1^)	MW_Cal_ (g mol^–1^)	Proposed Structure	MW_err_^a^ (%)
**3**	4-Me-Py	C_6_D_6_	4.36 × 10^–10^	1297	1314	[(Me-Py)_2_Zn_2_{O_2_P(OPh)_2_}_4_]	1.3
			1.21 × 10^–9^	218	93	4-Me-Py	–134
4	BiPy	CD_2_Cl_2_	7.37 × 10^–10^	907	719	[(BiPy)Zn{O_2_P(OPh)_2_}_2_]	–26
5	TMEDA	C_6_D_6_	5.73 × 10^–10^	803	680	[(TMEDA)Zn{O_2_P(OPh)_2_}_2_]	–18
6	PMDETA	C_6_D_6_	6.02 × 10^–10^	737	743	[(PMDETA)Zn{O_2_P(OPh)_2_}_2_]	0.8
7	Me_3_-TAC	C_6_D_6_	4.56 × 10^–10^	1200	1257	[(TAC)·Zn_2_{O_2_P(OPh)_2_}_4_]	4.5
			8.08 × 10^–10^	441	129	Me_3_-TAC	–242

a MW_err_ = [100 × (MW_cal_ – MW_det_)/MW_cal_].

**Figure 8:**
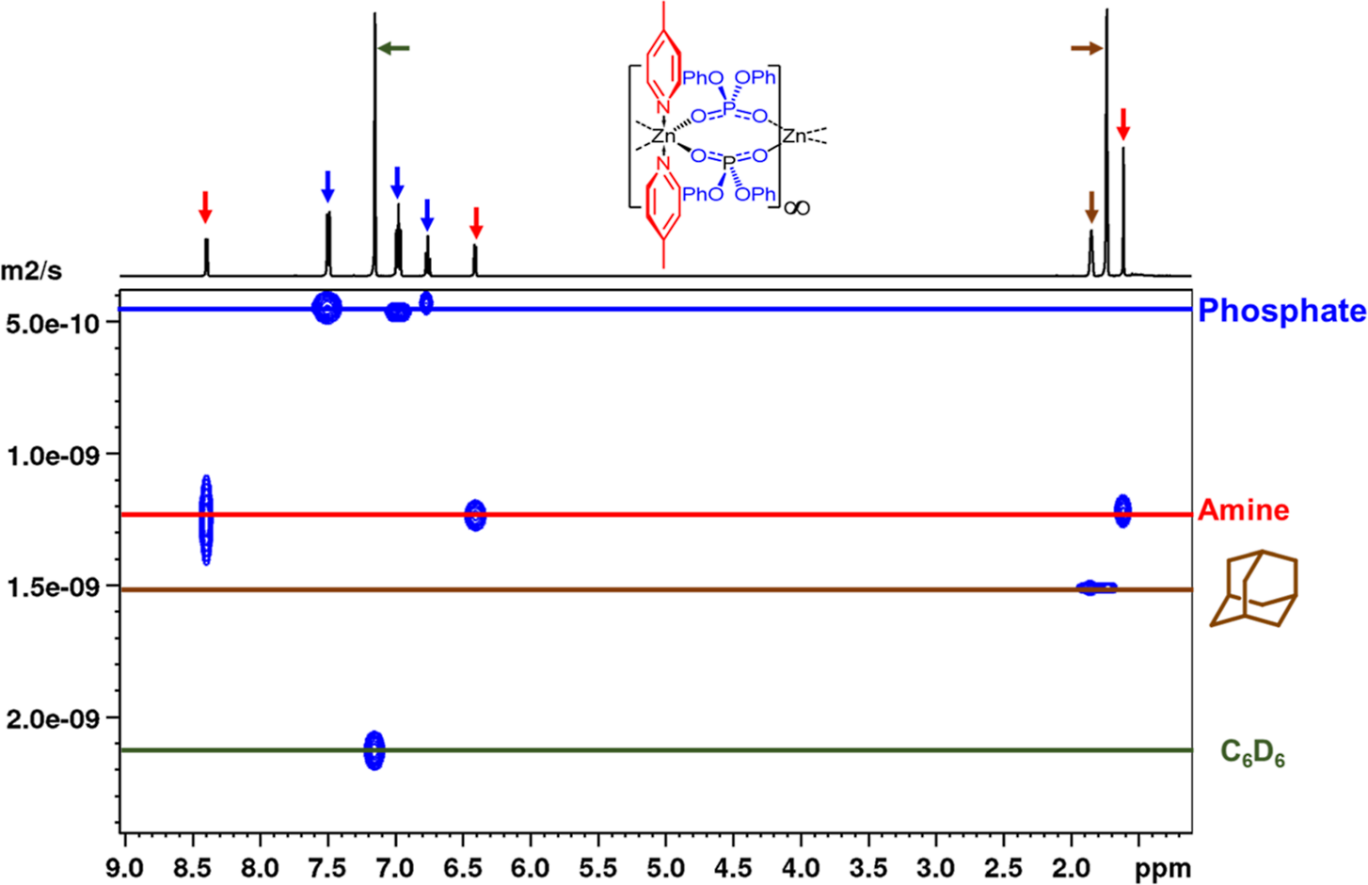
Plot showing the ^1^H DOSY NMR spectrum for compound **3** at 298 K.

**Scheme 3:**
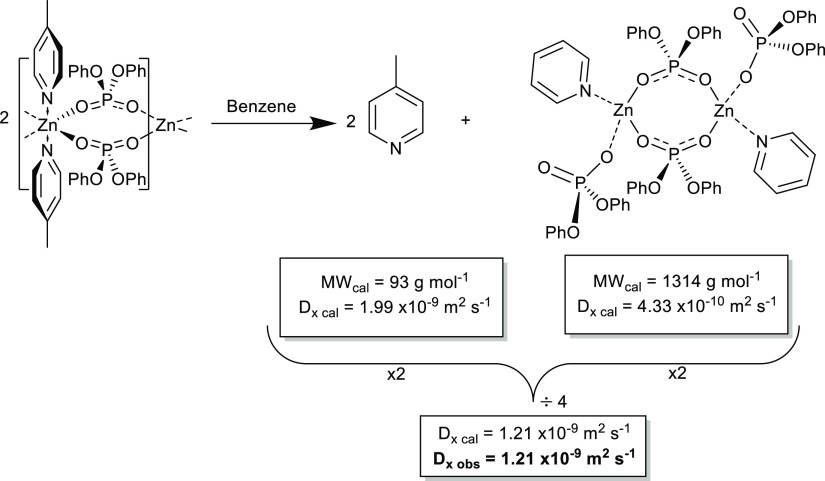
Proposed Solution State Zinc/Nitrogen–Ligand Exchange Process for **3** *D*_x cal_ values are determined from ECC data by ^1^H DOSY ECC-MW calculations, in benzene solution at 298 K.

The ^1^H NMR DOSY for compound **3**, as shown in Figure 8, clearly shows two separate diffusion coefficients, *D*_norm_ = 1.21 × 10^–9^ m^2^ s^–1^ and *D*_norm_ = 4.36 × 10^–10^ m^2^ s^–1^, respectively, indicating a break-up of polymeric **3** in solution. Accounting for both temperature and solvent viscosity, the observed (normalized) diffusion coefficients correspond to molecular weights (MW_det_) of 218 and 1297 g mol^–1^, respectively. While the slower diffusion coefficient is a good match for the bimetallic [(Me-Py)_2_·Zn_2_{O_2_P(OPh)_2_}_4_] (MW_cal_ = 1314 g mol^–1^, MW_err_ = 1.3%), the fast diffusion value corresponds to an apparent molecular weight of (MW_det_) of 218 g mol^–1^, which is not consistent with either [(Me-Py)_2_·Zn_2_{O_2_P(OPh)_2_}_4_] or free Me-Py (Scheme 3).

However, closer analysis of the data is consistent with a rapid nitrogen–ligand exchange process on the chemical shift time scale between [(Me-Py)_2_·Zn_2_{O_2_P(OPh)_2_}_4_] and two equivalents of free methylpyridine, as shown in Scheme 3, such that a normalized and weighted average of *D*_x cal_ = 1.21 × 10^–9^ m^2^ s^–1^ may be calculated (*cf.* an observed value of 1.21 × 10^–9^ m^2^ s^–1^, see in the Supporting Information).

To validate our hypothesis, excess methylpyridine (8 equiv) was added to a solution of **3** in benzene, with the expectation that the presence of the excess methylpyridine ligand would be reflected in an increase in the magnitude of the diffusion coefficient related to the methylpyridine resonances. As expected, two new normalized diffusion coefficients of *D*_norm_ = 1.80 × 10^–9^ m^2^ s^–1^ and *D*_norm_ = 4.72 × 10^–10^ m^2^ s^–1^ were observed. These values correspond to MW_det_ values of 116 g mol^–1^ (*cf.* 93 g mol^–1^ for Me-Py) MW_err_ = 25% for the methylpyridine containing species and a MW_det_ value of 1157 g mol^–1^ (*cf.* 1314 g mol^–1^) for [(Me-Py)_2_·Zn_2_(O_2_P(OPh)_2_)_4_] MW_err_ = 12%.

However, as a note of caution, we should highlight that as the diffusion coefficient of the phosphate containing species increases, this increase could also be consistent with more than one 4-Me-Py binding to each Zn center.

**Scheme 4:**
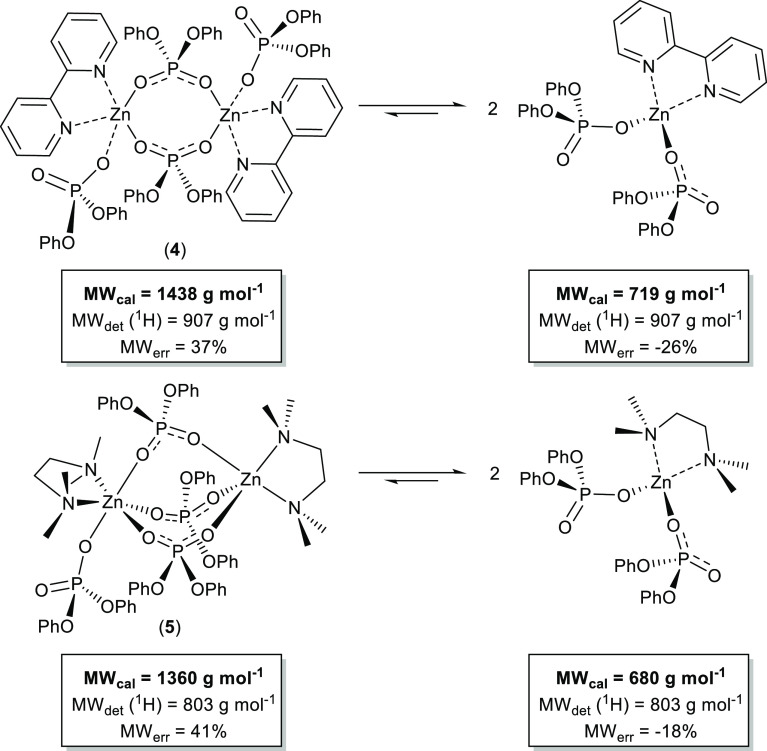
Formation of Monomeric Zinc Bis-diphenylphosphate Donor Base Adducts Form Dimers **4** and **5**

In the case of complexes **4**, **5**, and **6**, single diffusion signals are observed in the respective ^1^H DOSY experiments, consistent with either the presence of a single species in solution at room temperature or a time averaged signal. As can be seen from Table 7, the experimentally determined normalized diffusion coefficient for **6** (*D*_norm_ = 6.02 × 10^–10^ m^2^ s^–1^) corresponds to a determined molecular weight of 737 g mol^–1^ (MW_cal_ = 743 g mol^–1^, MW_err_ = 0.8%) consistent with a monometallic species, as observed in the solid state (Figure 5). In contrast, the experimentally determined diffusion coefficients for **4** (*D*_norm_ = 7.37 × 10^–10^ m^2^ s^–1^) and **5** (*D*_norm_ = 5.73 × 10^–10^ m^2^ s^–1^) produce MW_det_ (^1^H) values of 907 g mol^–1^ (**4**) and 803 g mol^–1^ (**5**), which are suggestive of a rapid (faster than the ^1^H NMR time scale) monomer–dimer equilibria, in which the equilibria lie significantly toward the formation of monomer at room temperature, as evidenced by the MW_err_ values (Scheme 4).

Variable temperature (223–313 K) ^1^H DOSY experiments were performed on **5** in *d*_8_-tol. The application of normalized diffusion coefficients renders the ^1^H DOSY experiment temperature independent, and changes in viscosity of the solvent with temperature were accounted for. Changing the NMR solvent from C_6_D_6_ to toluene-*d*_8_ results in a change in the experimentally determined normalized diffusion coefficients for **5** (298 K) from 5.73 × 10^–10^ m^2^ s^–1^(MW_det_ (^1^H) = 803 g mol^–1^) to 5.02 × 10^–10^ m^2^ s^–1^ (MW_det_ (^1^H) = 895 g mol^–1^) consistent with monomer dimer equilibria lying toward the dimeric species in the less polar solvent, toluene, *cf.* C_6_D_6_.^29^ Above 313 K, ^1^H DOSY experiments clearly show two different diffusion coefficients, consistent with the dissociation of the complex as indicated by the presence of separate TMEDA and a diphenylphosphate-containing species. Unfortunately, the solubility of the complex below 223 K precluded low-temperature experiments. At 313 K, a singular normalized diffusion coefficient (*D*_norm_ = 5.27 × 10^–10^ m^2^ s^–1^) corresponds to a determined molecular weight of 822 g mol^–1^ (MW_cal_ = 680 g mol^–1^) and is consistent with the monomer–dimer equilibria shifting toward the monomeric species at higher temperatures and to dimer formation at the low-temperature end of the range.

Table 8 shows the experimentally determined normalized diffusion coefficients at various temperatures alongside determined molecular weight values [MW_det_ (^1^H)], which when plotted against temperature shows a strong linear relationship (in the Supporting Information, Figure S14). Using the normalized diffusion coefficients, which are weighted mean averages of the composite monomer (*D*^M^_norm_) and dimer (*D*^D^_norm_) diffusion coefficients, we can in turn calculate an estimated equilibrium constant for the monomer dimer equilibrium at each individual temperature (see the Supporting Information). This can, in turn, be used to estimate both Δ*H* and Δ*S*, using the Van’t Hoff equation. Because the dimer is more stable enthalpically, but is disfavored entropically, the Δ*H* value should be positive in the dimer to monomer direction and the Δ*S* values should be positive in the dimer to monomer direction but negative in the monomer to dimer direction. Our observations fit these expectations, with the enthalpy [Δ*H* = −43.2 kJ mol^–1^ (±3.79)] and entropy [Δ*S* = 109 J mol^–1^ K^–1^ (±13.9)] of dimerization determined for **5** in toluene-*d*_8_. These data can be used to provide an approximate ΔG* value [ΔG* = 75.6 kJ mol^–1^ (±5.62) at 293 K] for the conversion of dimeric **5** into its monomeric form (in the Supporting Information, Figure S15).

**Table 8:** Observed and Normalized Diffusion Coefficients (*D*_obs_ and *D*_norm_), Molecular Weights Determined (by ^1^H DOSY) [(MW_det_ (^1^H)], and Calculated Equilibrium Constants (*K*_cal_) at Selected Temperatures for **5** (*d*_8_-tol)

Temp/K	*D*_obs_/m^2^ s^–1^	*D*_norm_/m^2^ s^–1^	MW_det_ (^1^H)/g mol^–1^	*K*_cal_/mol dm^–3^
313	7.6 × 10^–10^	5.27 × 10^–10^	822.8	2.54 × 10^–2^
298	5.73 × 10^–10^	5.02 × 10^–10^	894.9	1.19 × 10^–2^
283	4.3 × 10^–10^	4.93 × 10^–10^	922.6	9.29 × 10^–3^
268	2.9 × 10^–10^	4.4 × 10^–10^	1123.8	1.26 × 10^–3^
253	1.98 × 10^–10^	4.23 × 10^–10^	1200.2	4.67 × 10^–4^
238	1.4 × 10^–10^	4.13 × 10^–10^	1254.2	1.80 × 10^–4^
223	8.21 × 10^–11^	3.94 × 10^–10^	1357.5	7.74 × 10^–8^

Attempts to perform similar experiments on complex **4** in CD_2_Cl_2_, which we believe undergo comparable monomer dimer equilibria, were thwarted by solubility issues.

**Scheme 5:**
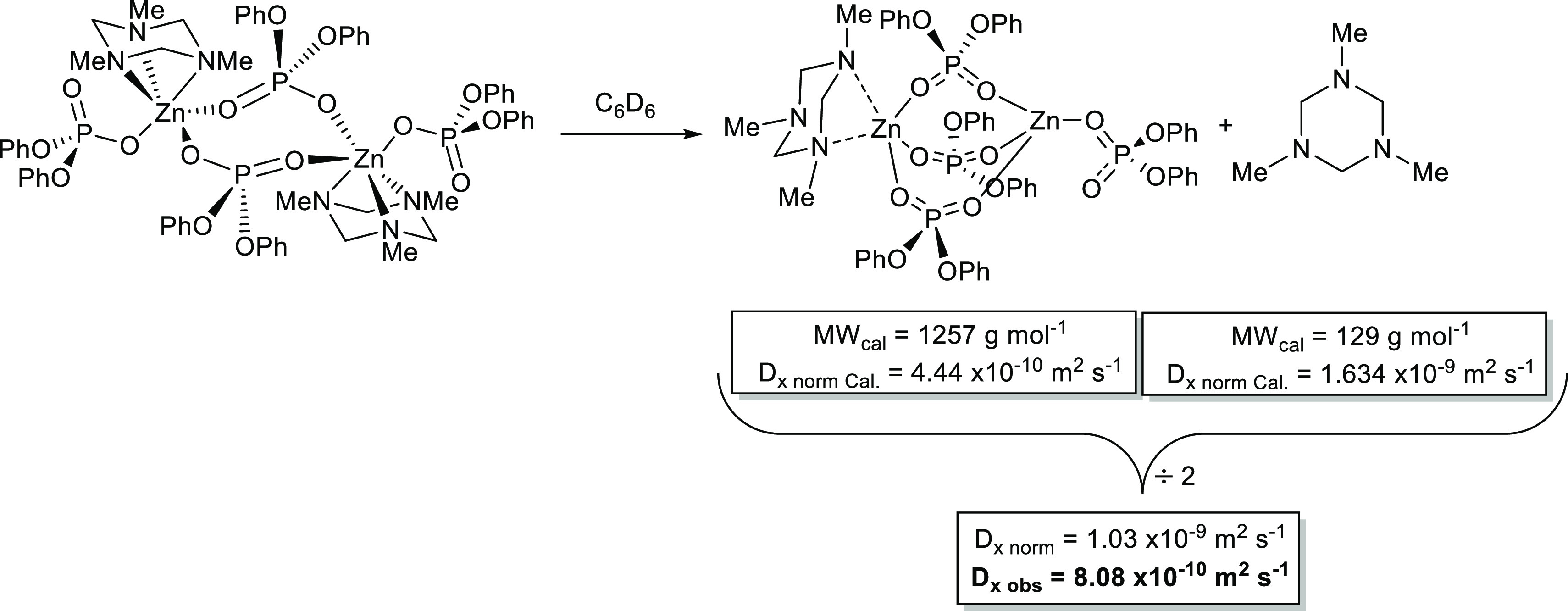
Proposed Solution Ligand Dissociation Observed in a C_6_D_6_ Solution of **7**, at 298 K

In contrast to complexes **4** and **5**, there is no evidence from ^1^H NMR studies of the putative monomer [(η^3^-Me_3_-TAC)Zn{O_2_P(OPh)_2_}_2_] expected from the de-oligomerization of **7** in solution. Rather, as can be seen clearly in Figure 7, ^1^H DOSY experiments at 298 K reveal two separate normalized diffusion coefficients (*D*_norm_ = 8.08 × 10^–9^ m^2^ s^–1^ and *D*_norm_ = 4.56 × 10^–10^ m^2^ s^–1^), consistent with a ligand dissociation similar to that observed for complex **3**. While the slower diffusion coefficient corresponds to a MW_det_ = 1200 g mol^–1^ and is in good agreement with the formation of a species such as [(Me_3_-TAC)·Zn_2_{O_2_P(OPh)_2_}_4_] (MW_cal_ = 1253 g mol^–1^; MW_err_ = 4.2%), the faster diffusion coefficient corresponds to a MW_det_ = 441 g mol^–1^ which is significantly different to the anticipated value of 129 g mol^–1^ for free Me_3_-TAC (Scheme 5).

As with complex **3**, a close inspection of the data is consistent with a rapid nitrogen–ligand exchange process on the chemical shift time scale between [(Me_3_-TAC)·Zn_2_{O_2_P(OPh)_2_}_4_] and free Me_3_-TAC, as shown in Scheme 5. For the two proposed species in solution, we can calculate normalized diffusion coefficients based on the molecular masses {*i.e.*, Me_3_-TAC; MW_cal_ = 129 g mol^–1^ and [(Me_3_-TAC)·Zn_2_{O_2_P(OPh)_2_}_4_] MW_cal_ = 1257 g mol^–1^} of 1.63 × 10^–9^ m^2^ s^–1^ and 4.44 × 10^–10^ m^2^ s^–1^ respectively. Assuming a 1:1 equilibria mix of free Me_3_-TAC and [(Me_3_-TAC)·Zn_2_{O_2_P(OPh)_2_}_4_], a calculated normalized diffusion coefficient (*D*_x norm_) of 1.03 × 10^–9^ m^2^ s^–1^ can be calculated, a value that is in close agreement with the experimentally observed diffusion coefficient of *D*_x norm(obs)_ = 8.08 × 10^–10^ m^2^ s^–1^ (Scheme 5). This discrepancy, whereby the observed diffusion coefficient is lower than that calculated for a 1:1 equilibrium, suggests that, on average slightly greater than one Me_3_-TAC molecule is bound to each Zn dimer.

## Conclusions

This work sets out to provide insights into the interaction between donor-bases and zinc dialkylphosphates that demonstrate potential for use in the development of zinc phosphate precursors. Our synthetic studies on di-phenyl-phosphate complexes of zinc have so far yielded a family of polymeric 1D structures, **1**, **2**, and **3**, dimeric systems, **4**, **5**, and **7**, as well and a mono-nuclear zinc *bis*-diphenylphosphate complex, **6**. The range of structural diversity observed here has also been observed in a variety of other zincophosphate networks.^7d,8a,30^

As such, combinations of {ZnO_4_} and {PO_4_} tetrahedra represent versatile building blocks for the formation of zincophosphate materials. While the structural diversity noted in other work has been attributed to the low bending potential associated with the Zn–O–P linkages, the Lewis basic nitrogen donor ligands used in this study play a large part in directing the solid-state structures identified.^17a^ Curiously, throughout our studies, we have never observed the formation of the oxo-centered tetranuclear cluster Zn_4_(μ^4^-O) prevalent in other studies.^17a,19^

Analysis of solid-state structures via SCXRD demonstrated that the coordination polymer formed in the absence of any amine can be broken up by multi-dentate amine ligands—with different ligands giving access to a range of dimeric and monomeric structures. Thus, knowing which aggregates are formed during a reaction is of high interest to develop better selectivity and higher yields and in our case understanding the form and function of precursors in solution-state applications of precursors, *e.g.*, aerosol-assisted chemical vapor deposition, is significantly beneficial. DOSY, which separates NMR signals according to the diffusion coefficient, has found increasing use as a means to identify species in solution. Here, using ^1^H DOSY NMR experiments, we have attempted to identify solution-state species via molecular weight determination.^3,4^ We have detailed the dynamic solution-state behavior of these systems and demonstrated the applicability of DOSY NMR experiments and Stalke’s ECCs to determine the molecular weights of complex dynamic systems beyond alkaline-metal organometallics.

## Experimental Section

### General Details

All manipulations of air- and moisture-sensitive compounds were carried out under an atmosphere of nitrogen or argon using standard Schlenk-line or glovebox techniques. Solvents were dried according to standard methods and collected by distillation. All ligands were purchased from commercial sources, and [Zn{N(SiMe_3_)_2_}_2_] was prepared according to the literature procedure.^31^

^1^H, ^13^C, and ^31^P NMR spectra were recorded on Bruker AVANCE 300 or 500 MHz FT-NMR spectrometers, as appropriate, in saturated solutions at room temperature. Chemical shifts are expressed in parts per million with respect to Me_4_Si (^1^H and ^13^C) or 85% H_3_PO_4_ in H_2_O (^31^P). DOSY experiments were carried out on a Bruker 500 MHz spectrometer, using a standard double attenuated echo sequence with longitudinal eddy current delay. Experiments were typically carried out with a gradient strength, ranging from 10 to 90% using smoothed square gradients, and with Δ and δ set to 50 and 2 ms, respectively. Data were processed using Bruker Dynamics Centre. IR spectra were recorded on a PerkinElmer Spectrum 100 ATR FT-IR spectrometer and analyzed using proprietary PerkinElmer software. Elemental analysis was conducted using an Exeter Analytical CE440 elemental analyzer. All samples were run in duplicates. While both direct and *in situ* methodologies for the synthesis of complexes **1**–**7** were used in this study, no disenable difference in the two methods could be found. Accordingly, only the *in situ* methodology is reported here.

#### Synthesis of [Zn{O_2_P(OPh)_2_}]∞ (**1**)

Diphenylphosphoric acid (0.500 g, 2.00 mmol) in THF (10 mL) was added to a stirring solution of [Zn{N(SiMe_3_)_2_}_2_] (0.386 g, 1.00 mmol) in THF (10 mL). After being left to stir for 30 min, the solvent was removed *in vacuo*. 10 mL of anhydrous toluene was added to the solid residue and heated to reflux. Hot filtration, through Celite, afforded a clear colorless filtrate. Removal of excess solvent under vacuum followed by storage at 4 °C yielded 0.324 g of crystals (yield = 57%). Elemental analysis C_24_H_20_O_8_P_2_Zn (expected): C: 50.92 (51.13) H: 3.665 (3.58) ^1^H NMR (C_6_D_6_, 500 MHz): δ 6.74 (2H, t, *J* = 7 Hz, OPh-*p*), 6.93 (4H, m, OPh-*m*), 7.29 (4H, d, *J* = 7 Hz, OPh-*o*). ^13^C NMR (C_6_D_6_, 125.7 MHz): δ 120.5 (OPh-*o*), 124.2 (OPh-*p*), 129.4 (OPh-*m*), 151.6 (OPh-1). ^31^P NMR (C_6_D_6_, 202 MHz): δ −13.9 IR ν_max_/cm^–1^ 1590 (aromatic C=C) 1485 (aromatic C=C), 1191 (P=O), 1099 (Zn–O–P).

#### Synthesis of [Py_2_·Zn{O_2_P(OPh)_2_}]∞ (**2**)

Diphenylphosphoric acid (0.500 g, 2.00 mmol) in toluene (10 mL) was added to a stirring solution of [Zn{N(SiMe_3_)_2_}_2_] (0.386 g, 1.00 mmol) and pyridine (0.158 g, 2.00 mmol) in THF (10 mL). After stirring for 30 min, the solvent was removed *in vacuo*. 5 mL of THF was added to the residue. Hot filtration through Celite afforded a clear colorless solution. Crystals were grown on standing at 4 °C. 0.177 g of **2** was isolated by filtration and washed with 5 mL of cold hexane and dried *in vacuo* (yield = 25%). Elemental analysis C_34_H_30_N_2_O_8_P_2_Zn (expected): C: 57.03 (56.57) H: 4.20 (4.19) N: 3.82 (3.88) ^1^H NMR (CD_2_Cl_2_, 500 MHz): δ 7.01 (m, 4H, OPh-*p*), 7.13 (m, 16H, OPh-o,*m*), 7.30 (m, 4H, Py-*m*), 7.77 (m, 2H, Py-*p*), 8.35 (m, 2H, Py-*o*). ^13^C NMR (CD_2_Cl_2_, 125.7 MHz): δ 120.6 (OPh-*o*), 124.6 (OPh-*p*), 124.9 (Py-*m*), 129.8 (OPh-*m*), 138.4 (Py-*p*), 149.6 (Py-*o*), 152.0 (OPh-1). ^31^P NMR (CD_2_Cl_2_, 202 MHz): δ −12.0. IR ν_max_/cm^–1^ 1590 (aromatic C=C) 1485 (aromatic C=C), 1190 (P=O), 1098 (Zn–O–P).

#### Synthesis of [Me-Py_2_·Zn{O_2_P(OPh)_2_}]∞ (**3**)

Complex **3** was synthesized in the same way as **2** using 4-methylpyridine (0.186 g, 2.00 mmol). THF was removed *in vacuo*, and 10 mL of anhydrous toluene was added to the solid residue and heated to reflux. Hot filtration, through Celite, afforded a clear colorless filtrate. Removal of excess solvent under vacuum followed by storage at 4 °C yielded 0.348 g of X-ray quality crystals (yield = 46%). Elemental analysis C_36_H_34_N_2_O_8_P_2_Zn (expected): C: 57.16 (57.65) H: 4.70 (4.57) N: 3.25 (3.74) ^1^H NMR (C_6_D_6_, 500 MHz): δ 1.62 (3H, s, Py*CH*_3_) 6.44 (d, *J* = 6 Hz, 2H, Py-*m*), 6.76 (m, 2H, OPh-*p*), 6.98 (m, 4H, OPh-m), 7.51 (m, 4H, OPh-*o*), 8.47 (m, 2H, Py-*o*). ^13^C NMR (C_6_D_6_, 125.7 MHz): δ 20.2 (Py*CH*_3_), 120.8 (OPh-*o*), 123.7 (Py-*m*), 124.9 (OPh-*p*), 129.2 (OPh-*m*), 148.5 (*Py*Me), 149.1 (Py-*o*), 152.5 (OPh-1). ^31^P NMR (C_6_D_6_, 202 MHz): δ −12.6. IR ν_max_/cm^–1^ 1592 (aromatic C=C) 1483 (aromatic C=C), 1201 (P=O), 1092 (Zn–O–P).

#### Synthesis of [bipy·Zn{O_2_P(OPh)_2_}]_2_ (**4**)

Complex **4** was synthesized in the same way as **2** using 2,2′-bipyridine (0.156 g, 1.00 mmol) in 10 mL of THF. 0.345 g of colorless crystals suitable for X-ray diffraction was grown from a concentrated dichloromethane solution layered with hexanes (yield = 48%). Elemental analysis C_34_H_28_N_2_O_8_P_2_Zn (expected): C: 56.38 (56.72) H: 3.94 (3.92) N: 3.95 (3.89) ^1^H NMR (CD_2_Cl_2_, 500 MHz): δ 7.03 (t, *J* = 7 Hz, 4H, OPh-*p*), 7.16 (m, 16H, OPh-*o*,*m*), 7.26 (*br.*m, 2H, *bipy*-2), 8.03 (*br.*m, 2H, *bipy*-3), 8.28 (*br.*m, 2H, bipy-4), 8.47 (*br.*m, 2H, *bipy*-1). ^13^C NMR (CD_2_Cl_2_, 125.7 MHz): δ 120.2 (OPh-*o*), 121.3 (bipy-4), 123.6 (OPh-*p*), 126.1 (bipy-2), 129.2 (OPh-*m*), 140.7 (bipy-3), 148.4 (bipy-5), 149.3 (bipy-1), 152.5 (OPh-1). ^31^P NMR (CD_2_Cl_2_, 202 MHz): δ −13.2. IR ν_max_/cm^–1^ 1588 (aromatic C=C) 1484 (aromatic C=C), 1203 (P=O), 1087 (Zn–O–P).

#### Synthesis of [TMEDA·Zn{O_2_P(OPh)_2_}]_2_ (**5**)

Complex **5** was synthesized in the same way as **2** using *N*,*N*,*N*′,*N*′-tetrametyhlethylenediamine (0.116 g, 1.00 mmol). 0.409 g of X-ray quality crystals was grown at −28 °C from the resulting solution (yield = 60%). Elemental analysis C_30_H_36_N_2_O_8_P_2_Zn (expected): C: 52.73 (52.99) H: 5.25 (5.34) N: 3.765 (4.12) ^1^H NMR (C_6_D_6_, 500 MHz): δ 1.87 (s, 4H, N*C*_2_*H*_4_) 2.12 (s, 12H, N*Me*), 6.82 (t, *J* = 7.5 Hz, 4H, OPh-*p*), 7.07 (m, *J* = 7.5 Hz, 8H, OPh-*m*), 7.58 (d, *J* = 7.5 Hz, 8H, OPh-*o*). ^13^C NMR (C_6_D_6_, 125.7 MHz): δ 46.4 (N*Me*), 56.0 (N*CH*_2_), 120.8 (OPh-*o*), 123.4 (OPh-*p*), 129.2 (OPh-*m*), 153.0 (OPh-1). ^31^P NMR (C_6_D_6_, 202 MHz): δ −14.0. IR ν_max_/cm^–1^ 1590 (aromatic C=C) 1489 (aromatic C=C), 1200 (P=O), 1087 (Zn–O–P).

#### Synthesis of [PMDETA·Zn{O_2_P(OPh)_2_}] (**6**)

Complex **6** was synthesized in the same way as **2** using *N*,*N*,*N*′,*N*″,*N*″-pentamethylethylenediamine (PMDETA) (0.173 g, 1.00 mmol). 0.522 g of X-ray quality crystals was grown at room temperature from the resulting solution (yield = 71%). Elemental analysis C_33_H_43_N_3_O_8_P_2_Zn (expected): C: 54.08 (53.78) H: 5.97 (5.88) N: 5.955 (5.70) ^1^H NMR (C_6_D_6_, 500 MHz): δ 1.41 (2H, *br* s), 1.69 (2H, *br* s), 2.06 (2H, *br* s), 2.21 (s, 3H, N*Me*), 2.40 (*br* s, 12H, N*Me*_2_), 6.85 (t, *J* = 7.5 Hz, 4H, OPh-*p*), 7.12 (m, 8H, OPh-*m*), 7.65 (d, *J* = 8 Hz, 8H, OPh-*o*). ^13^C NMR (C_6_D_6_, 125.7 MHz): δ 38.4 (N*Me*), 50.5 (N*CH*_2_), 51.8 (N*CH*_2_), 116.1 (OPh-*o*), 118.4 (OPh-*p*), 124.5 (OPh-*m*), 148.9 (OPh-1). ^31^P NMR (C_6_D_6_, 202 MHz): δ −12.4. IR ν_max_/cm^–1^ 1595 (aromatic C=C) 1487 (aromatic C=C), 1207 (P=O), 1096 (Zn–O–P).

#### Synthesis of [Me_3_-TAC·Zn{O_2_P(OPh)_2_}]_2_ (**7**)

Complex **7** was synthesized in the same way as **2** using trimethyl triazacyclohexane (Me_3_-TAC) (0.129 g, 1.00 mmol). 0.540 g of X-ray quality crystals was grown at −28 °C from the resulting solution (yield = 78%). Elemental analysis C_30_H_35_N_3_O_8_P_2_Zn (expected): C: 51.91 (52.00) H: 5.13 (5.09) N: 6.17 (6.06) ^1^H NMR (C_6_D_6_, 500 MHz): δ 2.03 (s, 9H, N*CH*_3_) 3.38 (s, 3H, N*CH*_2_N), 6.81 (t, *J* = 7 Hz, 4H, OPh-*p*), 7.05 (m, 8H, OPh-*m*), 7.53 (d, *J* = 7 Hz, 8H, OPh-*o*). ^13^C NMR (C_6_D_6_, 125.7 MHz): δ 34.0 (N*CH*_3_), 71.3 (N*CH*_2_N), 116.3 (OPh-*o*), 119.4 (OPh-*p*), 125.0 (OPh-*m*), 148.3 (OPh-1). ^31^P NMR (C_6_D_6_, 202 MHz): δ −12.8. IR ν_max_/cm^–1^ 1590 (aromatic C=C) 1489 (aromatic C=C), 1199 (P=O), 1096 (Zn–O–P).

### Single-Crystal X-ray Diffraction

Experimental details relating to the single-crystal X-ray crystallographic studies for compounds **1–7** are summarized in Table S2 (see the Supporting Information). All crystallographic data were collected at 150(2) K on a SuperNova, Dual, EosS2 diffractometer using radiation either Cu Kα (λ = 1.54184 Å) or Mo Kα (λ = 0.71073 Å). All structures were solved by direct methods followed by full-matrix least squares refinement on *F*^2^ using the WINGX-2014 suite of programs^32^ or OLEX2.^33^ All hydrogen atoms were included in idealized positions and refined using the riding model. Crystals were isolated from an argon-filled Schlenk flask and immersed under oil before being mounted onto the diffractometer. CCDC 2164451–2164457 contains the supplementary crystallographic data for this paper. These data can be obtained free of charge at www.ccdc.cam.ac.uk/conts/retrieving.html [or from the Cambridge Crystallographic Data Centre, 12, Union Road, Cambridge CB2 1EZ, UK; fax: +44-1223/336-033; E-mail: deposit@ccdc.cam.ac.uk].

## Supporting Information Available

^1^H, ^13^C{^1^H}, and DOSY NMR spectra and assignments of compounds **1**–**7**, alongside additional crystallographic data, including the molecular structure of compound **3** and crystal and structure refinement data for compounds **1–7** (PDF)
