# Supplementary material for: Exploration of Solid-State vs Solution-State Structure in Contact Ion Pair Systems: Synthesis, Characterization, and Solution-State Dynamics of Zinc Diphenyl Phosphate, [Zn{O2P(OPh)2}2], Donor-Base-Supported Complexes
Source: Inorg Chem. 2023 Mar 14;62(12):4770–85. doi: 10.1021/acs.inorgchem.2c03539 (PMC10052378; doi:10.1021/acs.inorgchem.2c03539)
Supplement: Supplementary file 1 — ic2c03539_si_001.pdf [file ic2c03539_si_001.pdf]

An Exploration of Solid-State vs Solution-State Structure in Contact Ion Pair Systems: Synthesis, Characterization and Solution State Dynamics of Zinc Diphenyl phosphate, [Zn{$O_2P(OPh)_2$}$_2$], Donor-base Supported Complexes

Andrew J. Straiton,[a] James D. Parish,[a,b], Joshua J. Smith,[b] John P. Lowe[c] and Andrew L. Johnson.*[a]

a. Department of Chemistry University of Bath, United Kingdom. BA2 7AY. E-mail: a.l.johnson@bath.ac.uk.

b. Infineum UK Ltd, Milton Hill Business & Technology Centre, Milton Hill, Abingdon OX13 6BB.

c. Material and Chemical Characterisation Facility (MC²), University of Bath, United Kingdom. BA2 7AY.

Table of Contents

Figure S1: A partially labelled plot of the asymmetric unit cell contents of **3**, [Zn(Me-Py)$_2${m-$O_2P(OPh)_2$}$_2$]∞, and a view of the polymeric chain 1D chain . Symmetry generated atoms are by the symmetry operators: (#) 1+x, y, z, and ($) x-1, y, z.....3

Table S1: Selected bond lengths (Å) and angles(°) for complex **3** 3

Table S2: Crystallographic data for the complexes **1** to **7**. 5

^{1}H DOSY NMR Experimental: 6

Scheme S1: Reference compounds used for validation of the ECC DOSY NMR method used. **A** was purchased from Sigma Aldrich and used without further purification. **B**[1], **C**[2,3] and **D**[4] were all prepared according to literature methods. 6

Figure S2: DOSY NMR spectrum of **3** in C_6D_6 at 298 K 7

Table S3: Diffusion data for compound **3** 7

Figure S3: DOSY NMR spectrum of **4** in CD_2Cl_2 at 298 K 8

Table S4: Diffusion data for compound **4** 8

Figure S4: DOSY NMR spectrum of **5** in C_6D_6 at 298 K 9

Table S5: Diffusion data for compound **5** 9

Figure S5: DOSY NMR spectrum of **6** in C_6D_6 at 298 K 10

Table S6: Diffusion data for compound **6** 10

Figure S6: DOSY NMR spectrum of **7** in C_6D_6 at 298 K 11

Table S7: Diffusion data for compound **7** 11

Figure S7: DOSY NMR spectrum of **5** in d_8-tol at 313 K. 12

Table S8: Diffusion data for compound **5** in d_8-tol at 313 K 12

Figure S8: DOSY NMR spectrum of **5** in d_8-tol at 298 K. 13

Table S9: Diffusion data for compound **5** in d_8-tol at 298 K 13

Figure S9: DOSY NMR spectrum of **5** in d_8-tol at 283 K. 14

Table S10: Diffusion data for compound **5** in d_8-tol at 283 K 14

Figure S10: DOSY NMR spectrum of **5** in d_8-tol at 268 K. 15

Table S11: Diffusion data for compound **5** in d_8-tol at 268 K 15

Figure S11: DOSY NMR spectrum of **5** in d_8-tol at 253 K. 16

Table S12: Diffusion data for compound **5** in d_8-tol at 253 K 16

Figure S12: DOSY NMR spectrum of **5** in d_8-tol at 238 K. The DOSY acquisition parameters were adjusted, such that d20 = 0.1, to increase signal attenuation at this temperature. 17

Table S13: Diffusion data for compound **5** in d_8-tol at 238 K 17

Figure S13: DOSY NMR spectrum of **5** in d_8-tol at 223 K. The DOSY acquisition parameters were adjusted, such that d20 = 0.1, to increase signal attenuation at this temperature. 18

Table S14: Diffusion data for compound **5** in d_8-tol at 223 K 18

Figure S14: A plot of MW_{det} against temperature for compound **5** 19

Figure S15: An Eyring plot for compound **5**, using equilibrium constants determined from DOSY NMR 19

Equilibria Calculations....................20

Figure S16: DOSY NMR spectrum of **3** in C_6D_6 with 4 excess equivalents of 4-Methyl pyridine at 298 K 22

Table S15: Diffusion data for compound **3** in C_6D_6 with 4 excess equivalents of 4-Methyl pyridine 22

Figure S17: DOSY NMR spectrum of diphenylphosphoric acid in C_6D_6 at 298 K....................23

Table S16: Diffusion data for diphenylphosphoric acid....................23

Figure S18: DOSY NMR spectrum of $[Zn(S_2CNEt_2)]$ in C_6D_6 at 298 K 24

Table S17: Diffusion data for $[Zn(S_2CNEt_2)]$ 24

Figure S19: DOSY NMR spectrum of $[Zn(\{OC_6H_2{}^tBu_2CH_2\}_2NC_2H_4NMe_2)]$ in C_6D_6 at 298 K....................25

Table S18: Diffusion data for $[Zn(\{OC_6H_2{}^tBu_2CH_2\}_2NC_2H_4NMe_2)]$ 25

Figure S20: DOSY NMR spectrum of $[Zn(2,2,6,6\text{-tetramethyl-3,5-heptanedione})_2]$ in d6-DMSO at 298 K 26

Table S19: Diffusion data for $[Zn(2,2,6,6\text{-tetramethyl-3,5-heptanedione})_2]$....................26

Supplementary References....................27

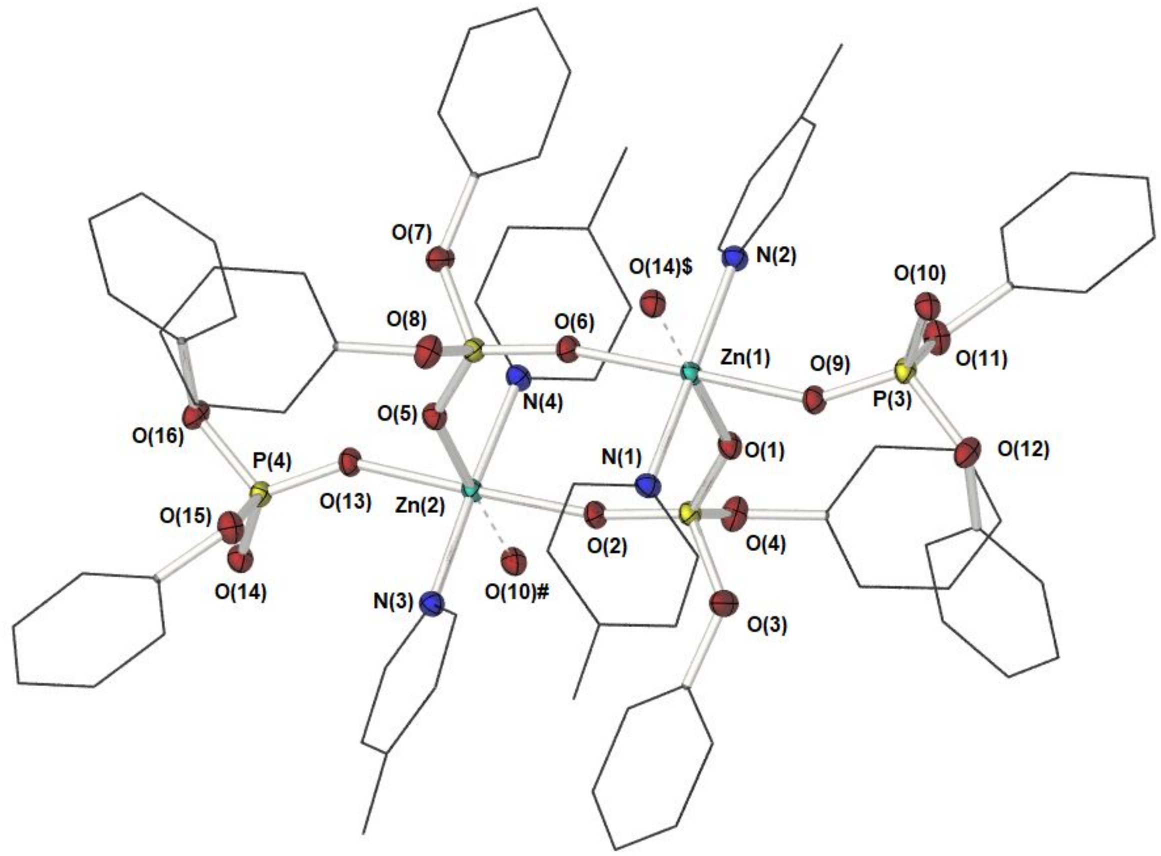

Figure S1: A partially labelled plot of the asymmetric unit cell contents of **3**, $[Zn(Me\text{-}Py)_2\{m\text{-}O_2P(OPh)_2\}_2]\infty$, and a view of the polymeric chain 1D chain . Symmetry generated atoms are by the symmetry operators: (#) 1+x, y, z, and ($) x-1, y, z.

Table S1: Selected bond lengths (Å) and angles(°) for complex **3**

Zn(1)-O(6)	2.0928(9)	Zn(2)-O(13)	2.0915(9)
Zn(1)-O(9)	2.0952(9)	Zn(2)-O(2)	2.1096(9)
Zn(1)-O(1)	2.1146(9)	Zn(2)-N(3)	2.1182(12)
Zn(1)-N(2)	2.1267(12)	Zn(2)-O(5)	2.1205(9)
Zn(1)-N(1)	2.1276(12)	Zn(2)-N(4)	2.1302(12)
Zn(1)-O(14)#1	2.1319(10)	Zn(2)-O(10)#2	2.1356(9)
O(6)-Zn(1)-O(9)	179.06(4)	N(2)-Zn(1)-O(14)#1	92.59(4)
O(6)-Zn(1)-O(1)	92.04(4)	N(1)-Zn(1)-O(14)#1	85.33(4)
O(9)-Zn(1)-O(1)	87.50(4)	O(13)-Zn(2)-O(2)	178.11(4)
O(6)-Zn(1)-N(2)	87.58(4)	O(13)-Zn(2)-N(3)	95.22(4)
		O(2)-Zn(2)-N(3)	85.76(4)
O(9)-Zn(1)-N(2)	93.22(4)	O(13)-Zn(2)-O(5)	86.95(4)
O(1)-Zn(1)-N(2)	88.80(4)	O(2)-Zn(2)-O(5)	91.46(4)
O(6)-Zn(1)-N(1)	92.82(4)	N(3)-Zn(2)-O(5)	88.76(4)
O(9)-Zn(1)-N(1)	86.40(4)	O(13)-Zn(2)-N(4)	87.11(4)
		O(2)-Zn(2)-N(4)	91.93(4)
O(1)-Zn(1)-N(1)	93.28(4)	N(3)-Zn(2)-N(4)	177.61(5)

N(2)-Zn(1)-N(1)	177.86(5)	O(5)-Zn(2)-N(4)	91.88(4)
O(6)-Zn(1)-O(14)#1	88.49(4)	O(13)-Zn(2)-O(10)#2	93.89(4)
O(9)-Zn(1)-O(14)#1	91.95(4)	O(2)-Zn(2)-O(10)#2	87.66(4)
O(1)-Zn(1)-O(14)#1	178.54(4)	N(3)-Zn(2)-O(10)#2	92.92(4)
		O(5)-Zn(2)-O(10)#2	178.05(4)
		N(4)-Zn(2)-O(10)#2	86.40(4)

Table S2: Crystallographic data for the complexes **1** to **7**.

Compound reference	**1**	**2**	**3**	**4**	**5**	**6**	**7**
Chemical formula	$C_{24}H_{20}O_8P_2Zn$	$C_{34}H_{30}N_2O_8P_2Zn\bullet C_4H_8O$	$C_{72}H_{68}N_4O_{16}P_4Zn_2$	$C_{68}H_{56}N_4O_{16}P_4Zn_2$	$C_{60}H_{72}N_4O_{16}P_4Zn_2$	$C_{33}H_{43}N_3O_8P_2Zn$	$C_{60}H_{70}N_6O_{16}P_4Zn_2$
Formula Mass	563.71	794.01	1499.92	1439.78	1359.83	737.01	1385.84
Crystal system	Trigonal	Triclinic	Monoclinic	Triclinic	Monoclinic	Monoclinic	Monoclinic
a/Å	12.81920(10)	10.45056(18)	10.51821(5)	14.2325(3)	23.2272(2)	11.73120(10)	12.41250(9)
b/Å	12.81920(10)	12.7930(2)	29.42952(16)	15.8583(3)	10.38810(10)	18.68300(10)	10.41639(8)
c/Å	12.66080(10)	14.2409(2)	23.02886(14)	17.2817(5)	27.1456(2)	15.94270(10)	24.44556(18)
α/°	90	102.9758(13)	90	115.112(2)	90	90	90
β/°	90	94.7201(13)	100.9262(5)	109.170(2)	104.0740(10)	96.3400(10)	94.6309(7)
γ/°	120	101.5354(14)	90	94.5830(19)	90	90	90
Unit cell volume/Å^3	1801.83(3)	1801.63(5)	6999.26(7)	3224.52(14)	6353.25(10)	3472.85(4)	3150.33(4)
Temperature/K	150(2)	150(2)	150(2)	150(2)	150(2)	150(2)	150(2)
Space group	$P3_2$	$P\bar{1}$	*P*21/*c*	$P\bar{1}$	*P*21/*c*	*P*21/*c*	*P*21/*c*
Z	3	2	4	2	4	4	2
Radiation type	Cu Kα	Cu Kα	Cu Kα	Cu Kα	Cu Kα	Cu Kα	Cu Kα
Absorption coefficient, μ/mm^{-1}	3.106	2.284	2.296	2.469	2.462	2.303	2.504
No. of reflections measured	22579	35030	91269	31040	78845	34378	37110
No. of independent reflections	3899	7169	13953	12792	12729	6942	6440
R_{int}	0.0483	0.0443	0.0281	0.0220	0.0349	0.0309	0.0267
Final R_1 values ($I > 2\sigma(I)$)	0.0368	0.0317	0.0290	0.0285	0.0264	0.0289	0.0251
Final $wR(F^2)$ values ($I > 2\sigma(I)$)	0.0882	0.0782	0.0742	0.0750	0.0674	0.0766	0.0683
Final R_1 values (all data)	0.0369	0.0378	0.0334	0.0323	0.0284	0.0306	0.0273
Final $wR(F^2)$ values (all data)	0.0885	0.0815	0.0770	0.0777	0.0687	0.0779	0.0698
Goodness of fit on F^2	1.060	1.035	1.066	1.026	1.037	1.067	1.050
Flack parameter	-0.02(3)	-	-	-	-	-	-
CCDC number	2164451	2164452	2164453	2164454	2164455	2164456	2164457

^{1}H DOSY NMR Experimental:

Convection compensated DOSY NMR spectra were acquired on a Bruker ProPulse 500 MHz spectrometer using the Bruker double stimulated echo pulse sequence "dstebpgp3s". Data was collected using eight gradient strengths between 10 and 90 % of full power, using smooth square shaped gradients, Δ = 50 ms, δ = 2 ms, and 16 scans at each gradient strength. Data was processed using Bruker Dynamics Centre software. Compound diffusion coefficients were obtained by taking an average of the diffusion coefficients of each resonance.

Spectra were all obtained at 15 mM, with each sample containing an internal adamantane reference (15 mM). Molecular masses were estimated using diffusion coefficients determined relative to adamantane and external calibration curves developed by Stalke and coworkers – as discussed in the main body of the text.

Initial experiments using four reference compounds with known molecular structures similar to the systems being studied within this work (Scheme S1). Figures S10-S13 show the diffusion spectra obtained. This determined that the "merged" calibration curve developed by Stalke and coworkers was the most appropriate for use in this work, returning estimated masses as shown in Table 6. This is in contrast to work on Li based organometallic species, where the diffused spheres and elipsoids (DSE) calibration curve offered more appropriate estimations. We hypothesise that this discrepancy is as a result of the increased electron density of the Zn and P centres within this study.

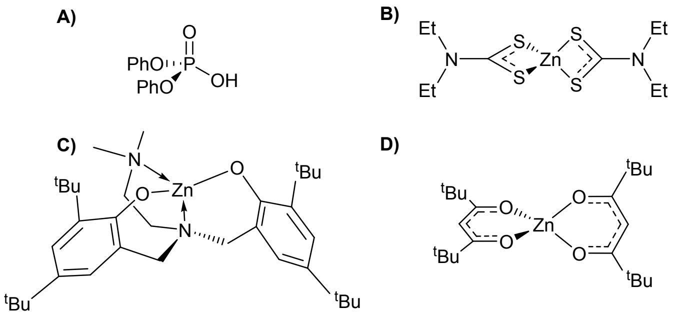

Scheme S1: Reference compounds used for validation of the ECC DOSY NMR method used. **A** was purchased from Sigma Aldrich and used without further purification. **B**[1], **C**[2,3] and **D**[4] were all prepared according to literature methods.

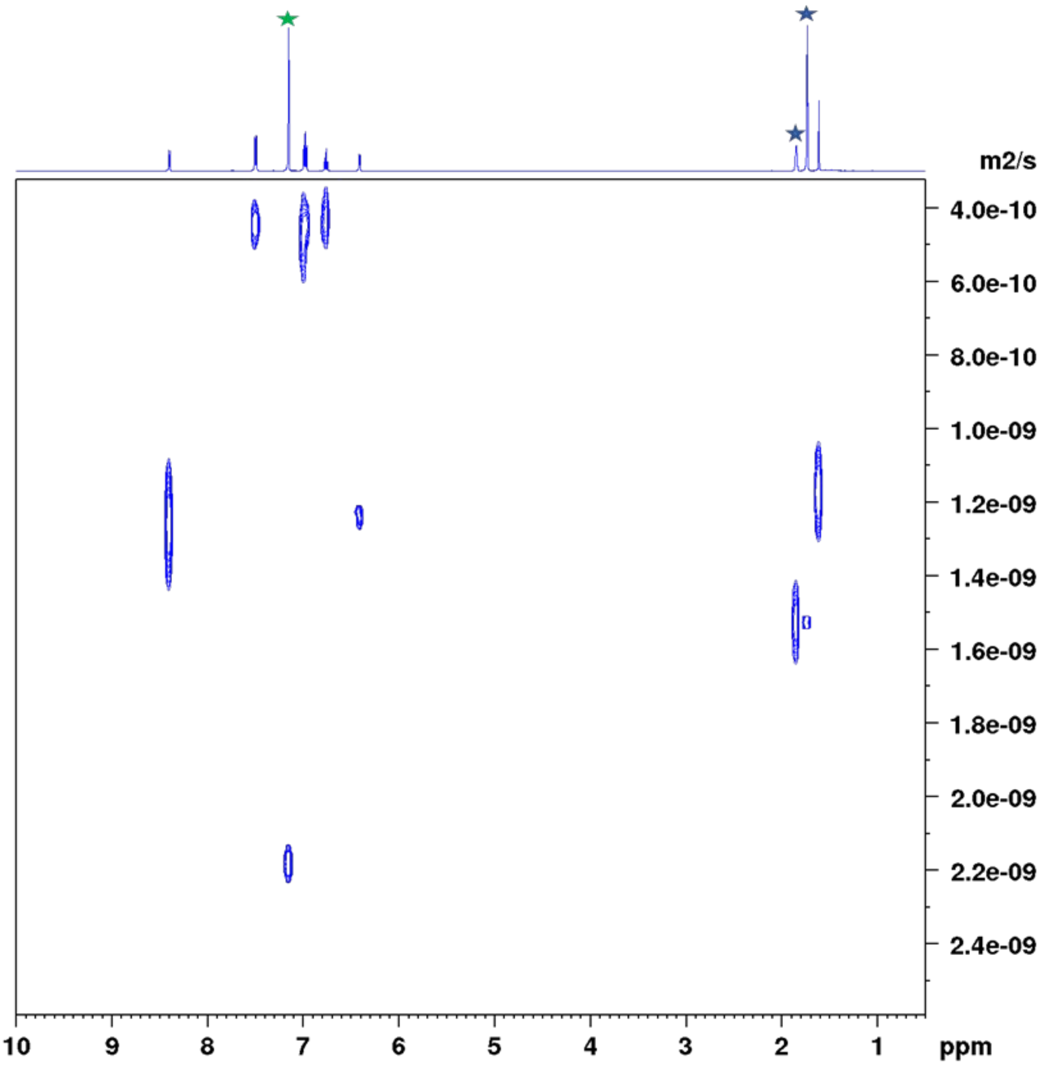

Figure S2: DOSY NMR spectrum of **3** in C_6D_6 at 298 K. Residual protio solvent (green) and Adamantane (blue) (internal standard) are marked respectively.

Table S3: Diffusion data for compound **3**

	**Phosphate**	**Base**	**Adamantane**
**Observed Diffusion Coefficient (m^2 s^{-1})**	4.15×10^{-10}	1.15×10^{-9}	1.50×10^{-9}
**Normalised Diffusion Coefficient (m^2 s^{-1})**	4.36×10^{-10}	1.21×10^{-9}	
**MW_{cal} (g mol^{-1})**	1314 [a]	93 [b]	
**MW_{det} (g mol^{-1})**	1297	218	
**MW_{err}**	1.3 %	-134 %	

[a] MW_{cal} assumes a structure of $[(Me\text{-}Py)_2Zn_2\{O_2P(OPh)_2\}_4]$

[b] MW_{cal} assumes a structure of [4-MePy]

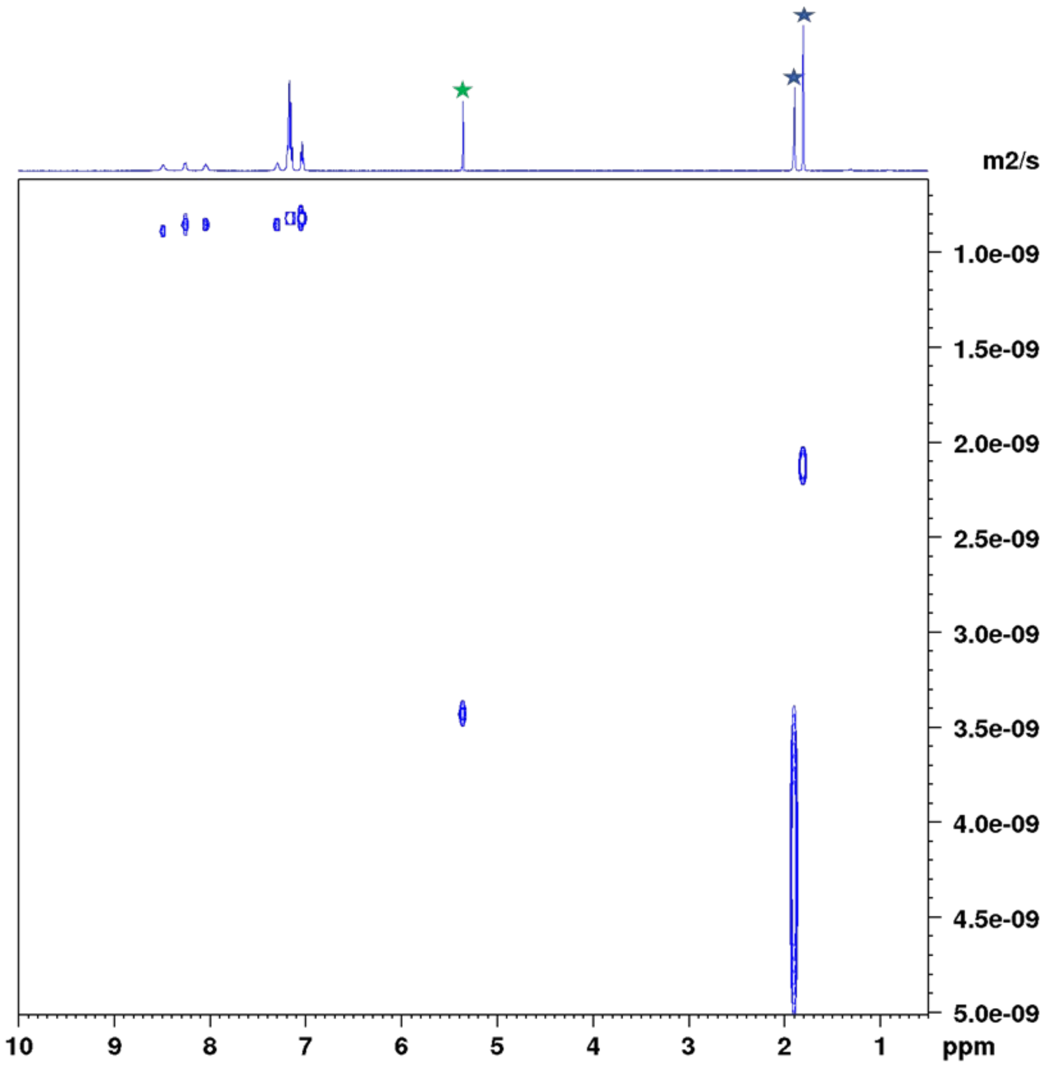

Figure S3: DOSY NMR spectrum of **4** in CD_2Cl_2 at 298 K. Residual protio solvent (green) and Adamantane (blue) (internal standard) are marked respectively.

Table S4: Diffusion data for compound **4**

	**Complex**	**Adamantane**
**Observed Diffusion Coefficient (m^2 s^{-1})**	7.68×10^{-10}	2.06×10^{-9}
**Normalised Diffusion Coefficient (m^2 s^{-1})**	7.37×10^{-10}	
**MW_{cal} (g mol^{-1})**	719 [a]	
**MW_{det} (g mol^{-1})**	907	
**MW_{err}**	-26 %	

[a] MW_{cal} assumes a monomeric structure of $[Zn\{O_2P(OPh)_2\}_2\{bipy\}]$

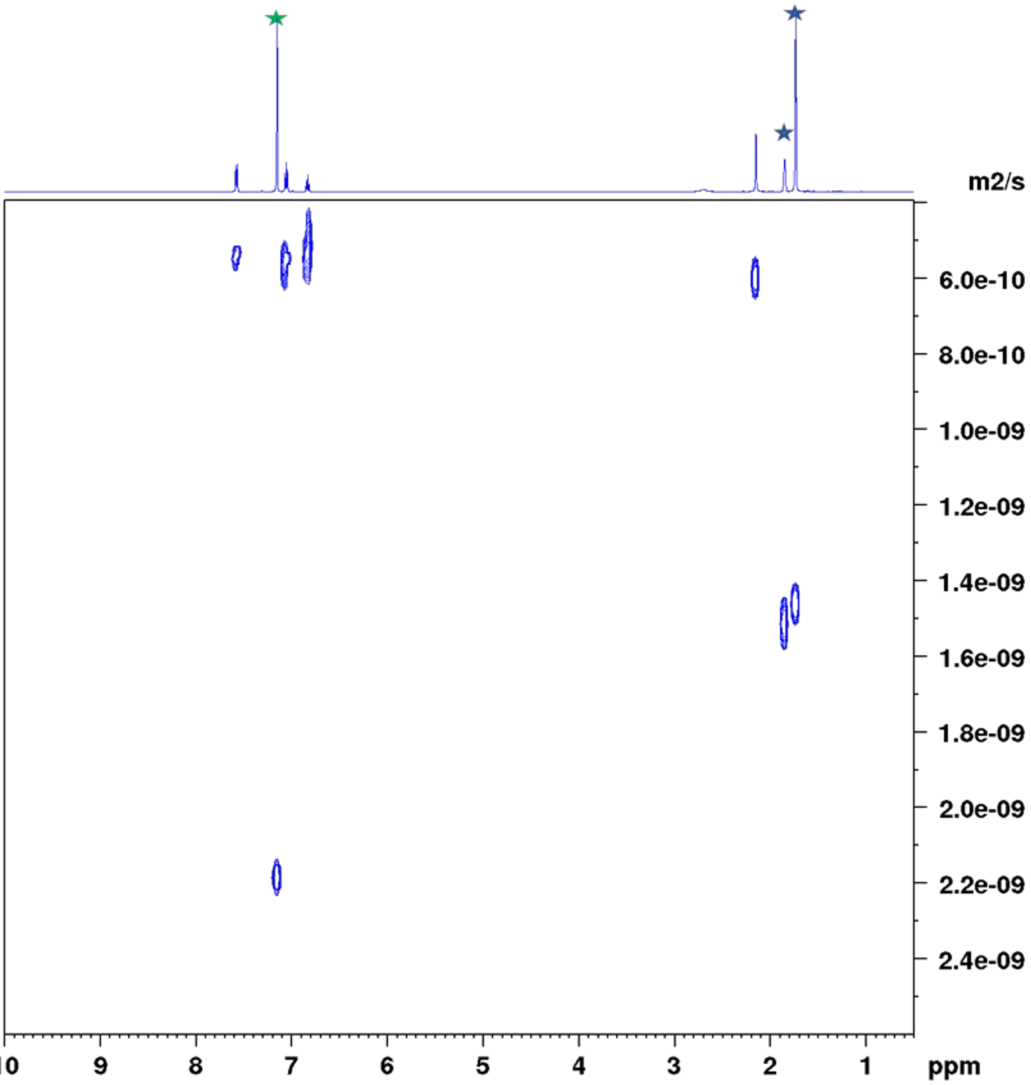

Figure S4: DOSY NMR spectrum of **5** in C_6D_6 at 298 K. Residual protio solvent (green) and Adamantane (blue) (internal standard) are marked respectively.

Table S5: Diffusion data for compound **5**

	**Complex**	**Adamantane**
**Observed Diffusion Coefficient (m^2 s^{-1})**	5.33×10^{-10}	1.47×10^{-9}
**Normalised Diffusion Coefficient (m^2 s^{-1})**	5.73×10^{-10}	
**MW_{cal}^{a} (g mol^{-1})**	680 [a]	
**MW_{det} (g mol^{-1})**	803	
**MW_{err}**	-18 %	

[a] MW_{cal} assumes a monomeric structure of $[Zn\{O_2P(OPh)_2\}_2\{TMEDA\}]$

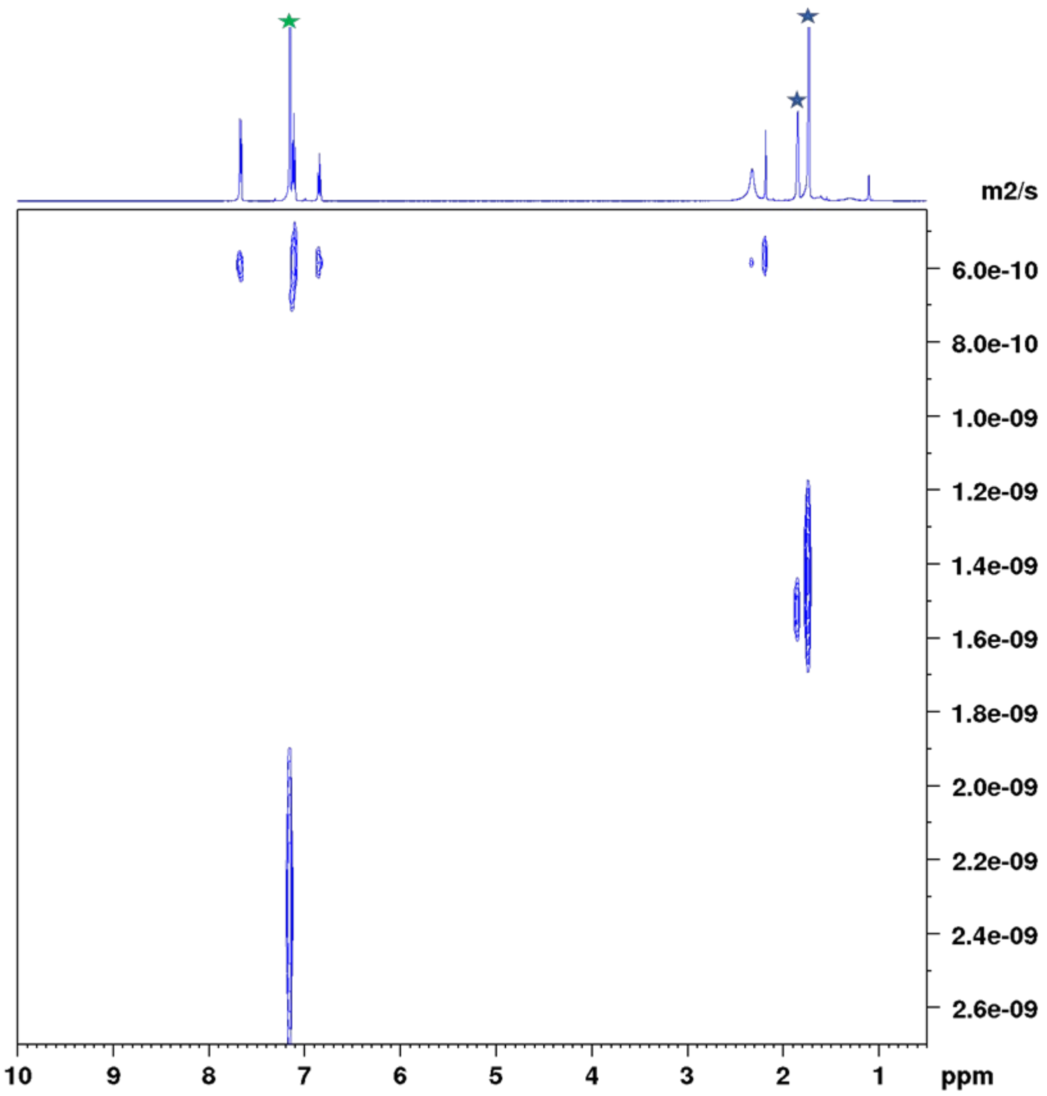

Figure S5: DOSY NMR spectrum of **6** in C_6D_6 at 298 K. Residual protio solvent (green) and Adamantane (blue) (internal standard) are marked respectively.

Table S6: Diffusion data for compound **6**

	**Complex**	**Adamantane**
**Observed Diffusion Coefficient (m^2 s^{-1})**	5.54×10^{-10}	1.45×10^{-9}
**Normalised Diffusion Coefficient (m^2 s^{-1})**	6.02×10^{-10}	
**MW_{cal} (g mol^{-1})**	743 [a]	
**MW_{det} (g mol^{-1})**	737	
**MW_{err}**	1 %	

[a] MW_{cal} assumes a structure as observed in the solid state of $[Zn\{O_2P(OPh)_2\}_2\{PMDETA\}]$

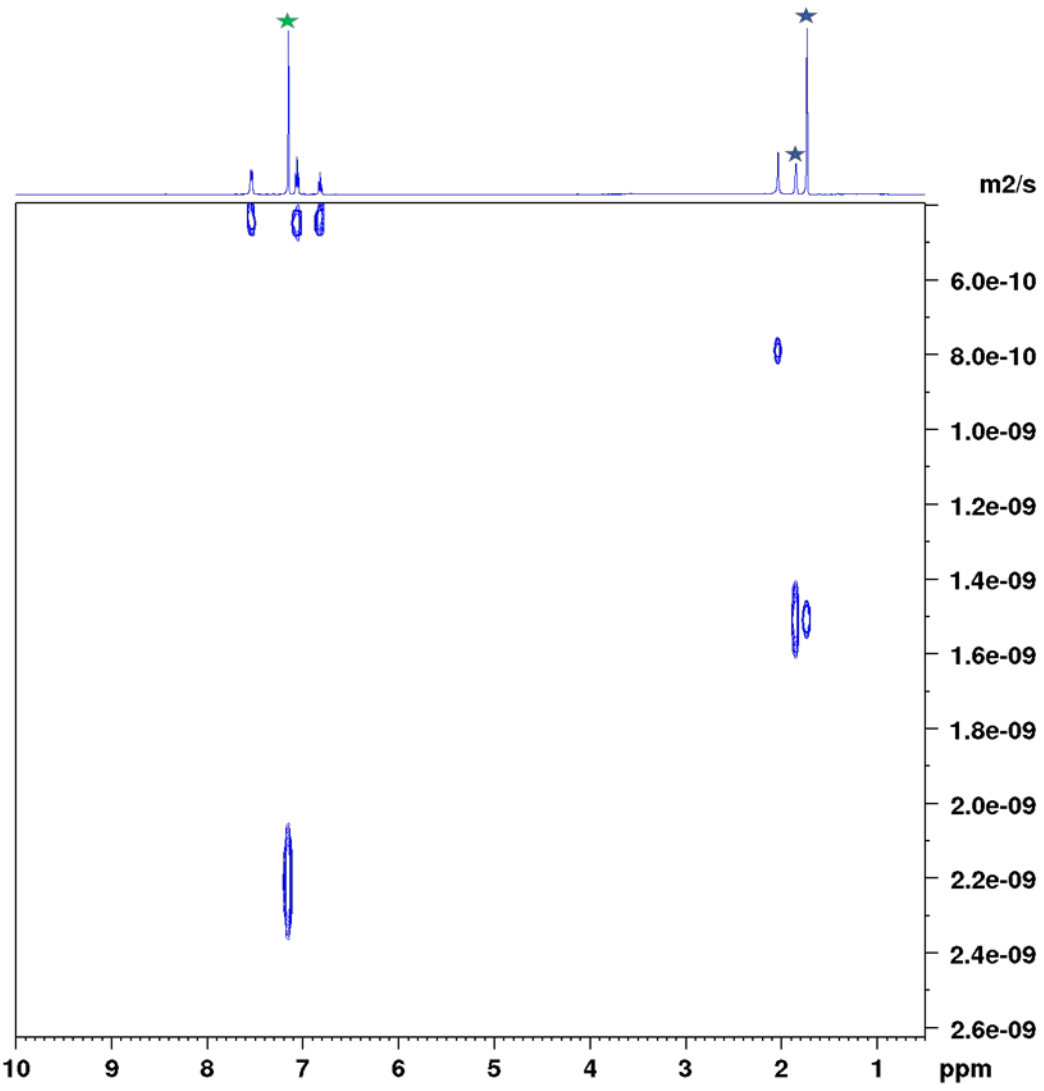

Figure S6: DOSY NMR spectrum of **7** in C_6D_6 at 298 K. Residual protio solvent (green) and Adamantane (blue) (internal standard) are marked respectively.

Table S7: Diffusion data for compound **7**

	**Phosphate**	**Base**	**Adamantane**
**Observed Diffusion Coefficient (m^2 s^{-1})**	4.31 x 10^{-10}	7.64 x 10^{-10}	1.49 x 10^{-9}
**Normalised Diffusion Coefficient (m^2 s^{-1})**	4.56 x 10^{-10}	8.08 x 10^{-10}	
**MW_{cal} (g mol^{-1})**	1257 [a]	129 [b]	
**MW_{det} (g mol^{-1})**	1200	441	
**MW_{err}**	4.5 %	-242 %	

[a] MW_{cal} assumes a structure of [(TAC)Zn_2{$O_2P(OPh)_2$}$_4$]

[b] MW_{cal} assumes a structure of [Me_3-{TAC}]

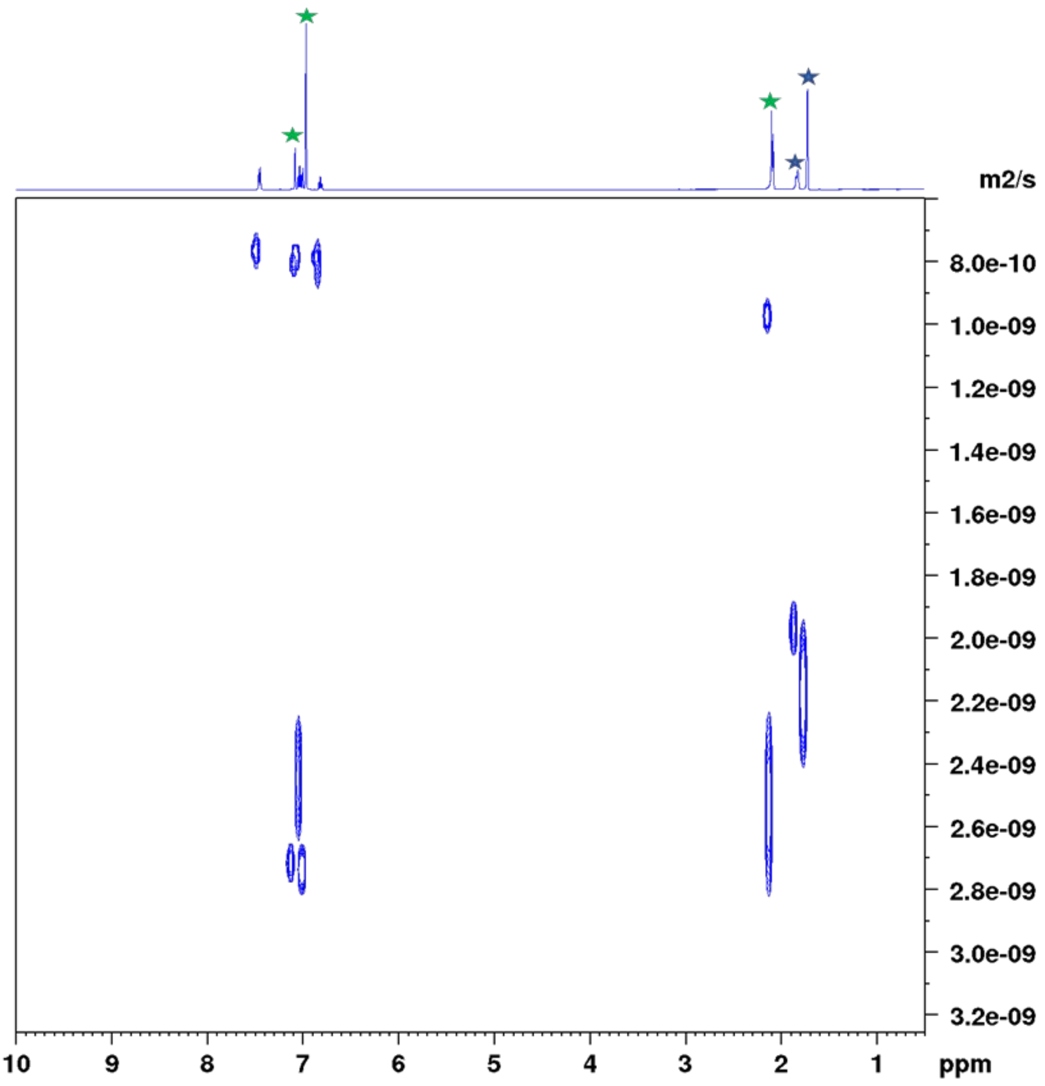

Figure S7: DOSY NMR spectrum of **5** in d_8-tol at 313 K. Residual protio solvent (green) and Adamantane (blue) (internal standard) are marked respectively.

Table S8: Diffusion data for compound **5** in d_8-tol at 313 K

	**Complex**	**Adamantane**
**Observed Diffusion Coefficient (m^2 s^{-1})**	7.60 x 10^{-10}	2.06 x 10^{-9}
**Normalised Diffusion Coefficient (m^2 s^{-1})**	5.27 x 10^{-10}	
**MW_{det} (g mol^{-1})**	823	
**K (mol dm^{-3})**	2.54 x 10^{-2}	

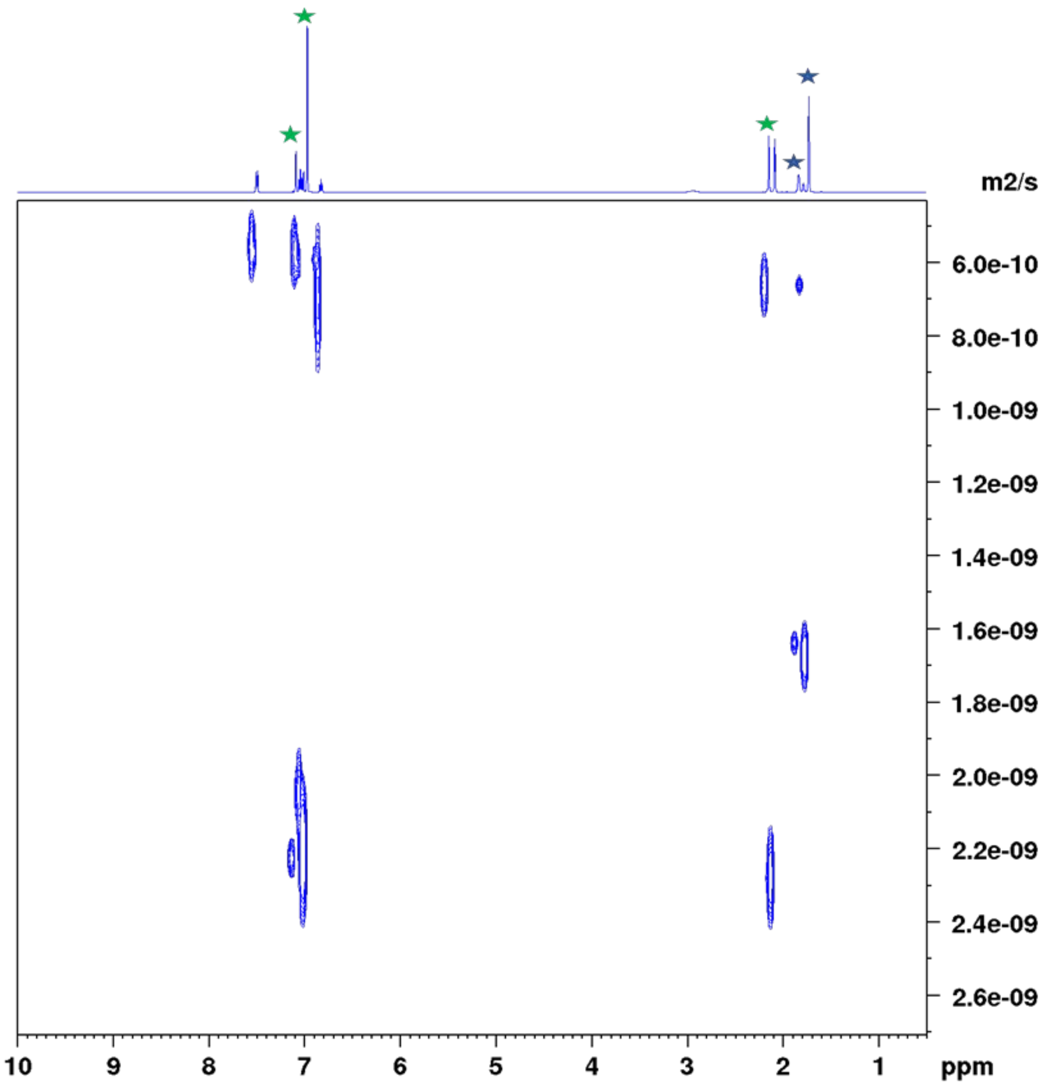

Figure S8: DOSY NMR spectrum of **5** in d_8-tol at 298 K. Residual protio solvent (green) and Adamantane (blue) (internal standard) are marked respectively.

Table S9: Diffusion data for compound **5** in d_8-tol at 298 K

	**Complex**	**Adamantane**
**Observed Diffusion Coefficient (m^2 s^{-1})**	5.73 x 10^{-10}	1.63 x 10^{-9}
**Normalised Diffusion Coefficient (m^2 s^{-1})**	5.02 x 10^{-10}	
**MW_{det} (g mol^{-1})**	895	
**K (mol dm^{-3})**	1.19 x 10^{-2}	

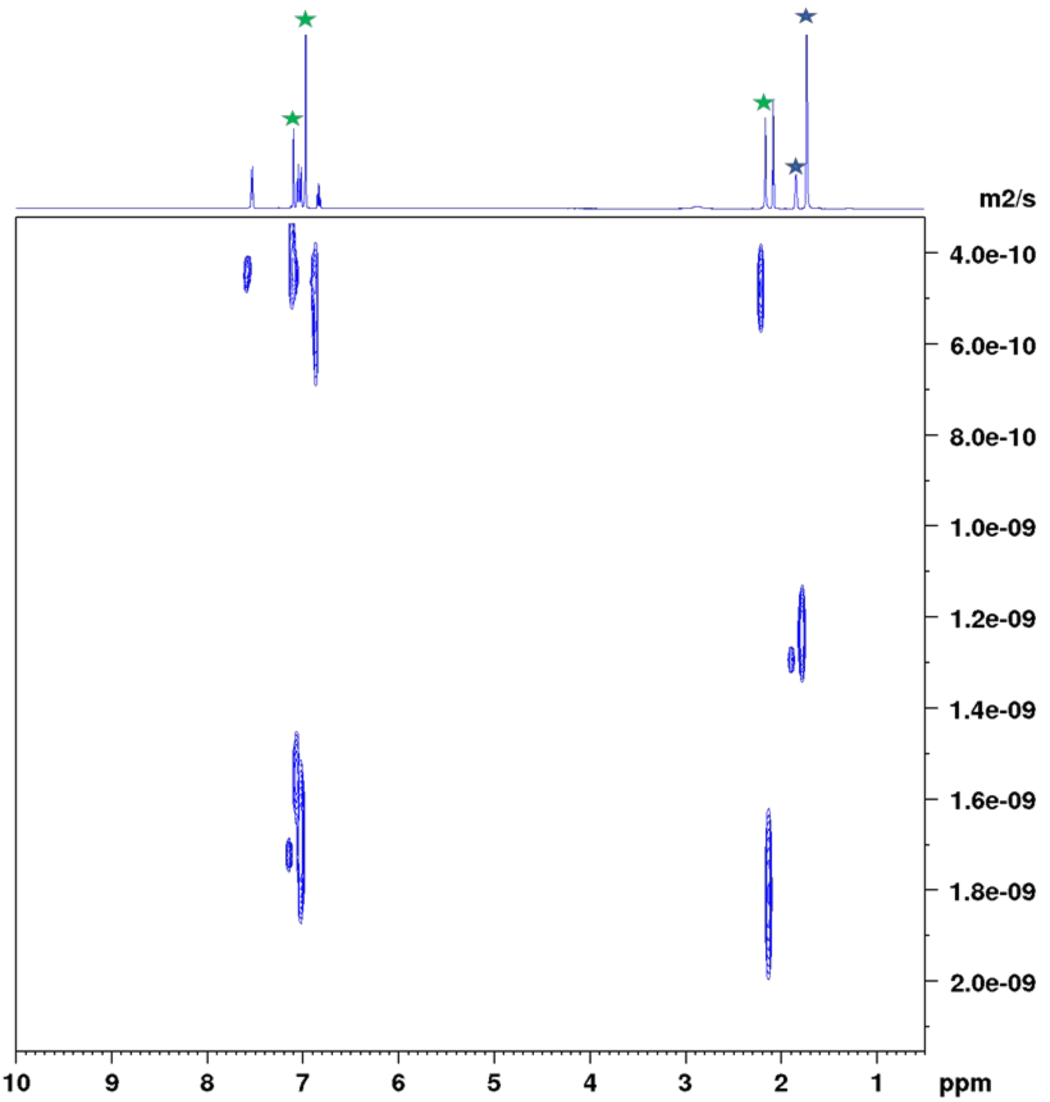

Figure S9: DOSY NMR spectrum of **5** in d_8-tol at 283 K. Residual protio solvent (green) and Adamantane (blue) (internal standard) are marked respectively.

Table S10: Diffusion data for compound **5** in d_8-tol at 283 K

	**Complex**	**Adamantane**
**Observed Diffusion Coefficient (m^2 s^{-1})**	4.30×10^{-10}	1.25×10^{-9}
**Normalised Diffusion Coefficient (m^2 s^{-1})**	4.93×10^{-10}	
**MW_{det} (g mol^{-1})**	923	
**K (mol dm^{-3})**	9.29×10^{-3}	

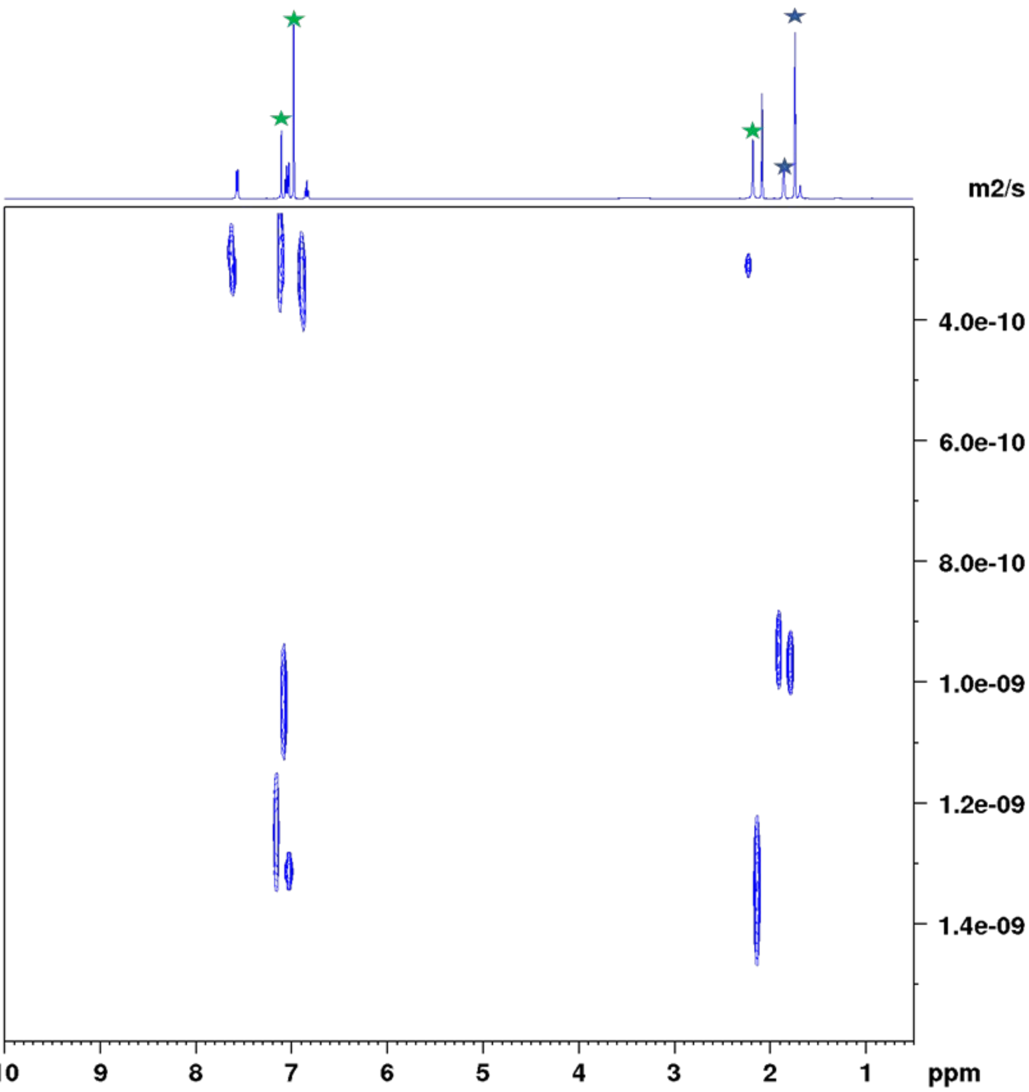

Figure S10: DOSY NMR spectrum of **5** in d_8-tol at 268 K. Residual protio solvent (green) and Adamantane (blue) (internal standard) are marked respectively.

Table S11: Diffusion data for compound **5** in d_8-tol at 268 K

	**Complex**	**Adamantane**
**Observed Diffusion Coefficient ($m^2 s^{-1}$)**	2.90×10^{-10}	9.43×10^{-10}
**Normalised Diffusion Coefficient ($m^2 s^{-1}$)**	4.40×10^{-10}	
**MW_{det} (g mol^{-1})**	1124	
**K (mol dm^{-3})**	1.26×10^{-3}	

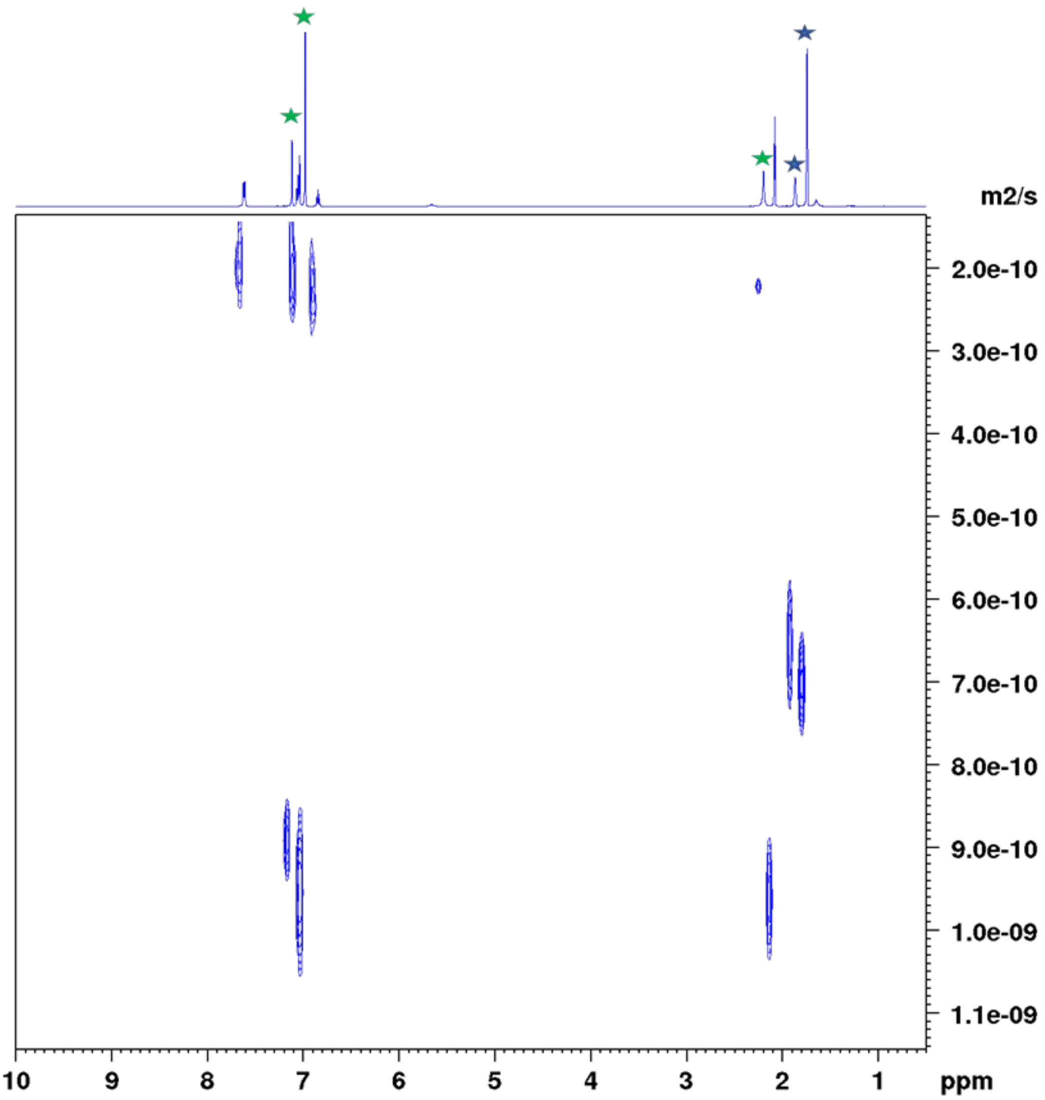

Figure S11: DOSY NMR spectrum of **5** in d_8-tol at 253 K. Residual protio solvent (green) and Adamantane (blue) (internal standard) are marked respectively.

Table S12: Diffusion data for compound **5** in d_8-tol at 253 K

	**Complex**	**Adamantane**
**Observed Diffusion Coefficient (m^2 s^{-1})**	1.98 x 10^{-10}	6.69 x 10^{-10}
**Normalised Diffusion Coefficient (m^2 s^{-1})**	4.23 x 10^{-10}	
**MW_{det} (g mol^{-1})**	1200	
**K (mol dm^{-3})**	4.67 x 10^{-4}	

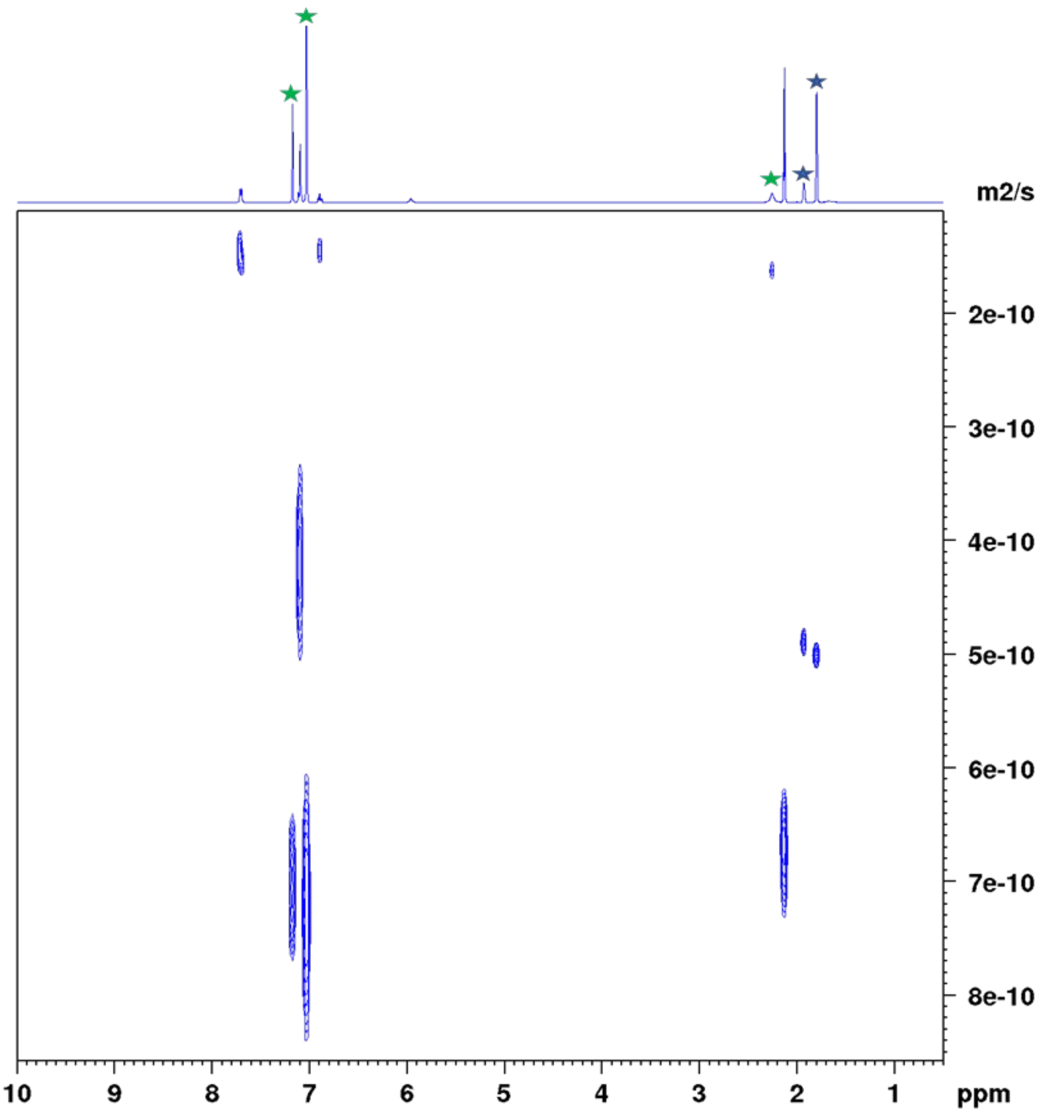

Figure S12: DOSY NMR spectrum of **5** in d_8-tol at 238 K. The DOSY acquisition parameters were adjusted, such that d20 = 0.1, to increase signal attenuation at this temperature. Residual protio solvent (green) and Adamantane (blue) (internal standard) are marked respectively.

Table S13: Diffusion data for compound **5** in d_8-tol at 238 K

	**Complex**	**Adamantane**
**Observed Diffusion Coefficient (m^2 s^{-1})**	1.40 x 10^{-10}	4.86 x 10^{-10}
**Normalised Diffusion Coefficient (m^2 s^{-1})**	4.13 x 10^{-10}	
**MW_{det} (g mol^{-1})**	1254	
**K (mol dm^{-3})**	1.80 x 10^{-4}	

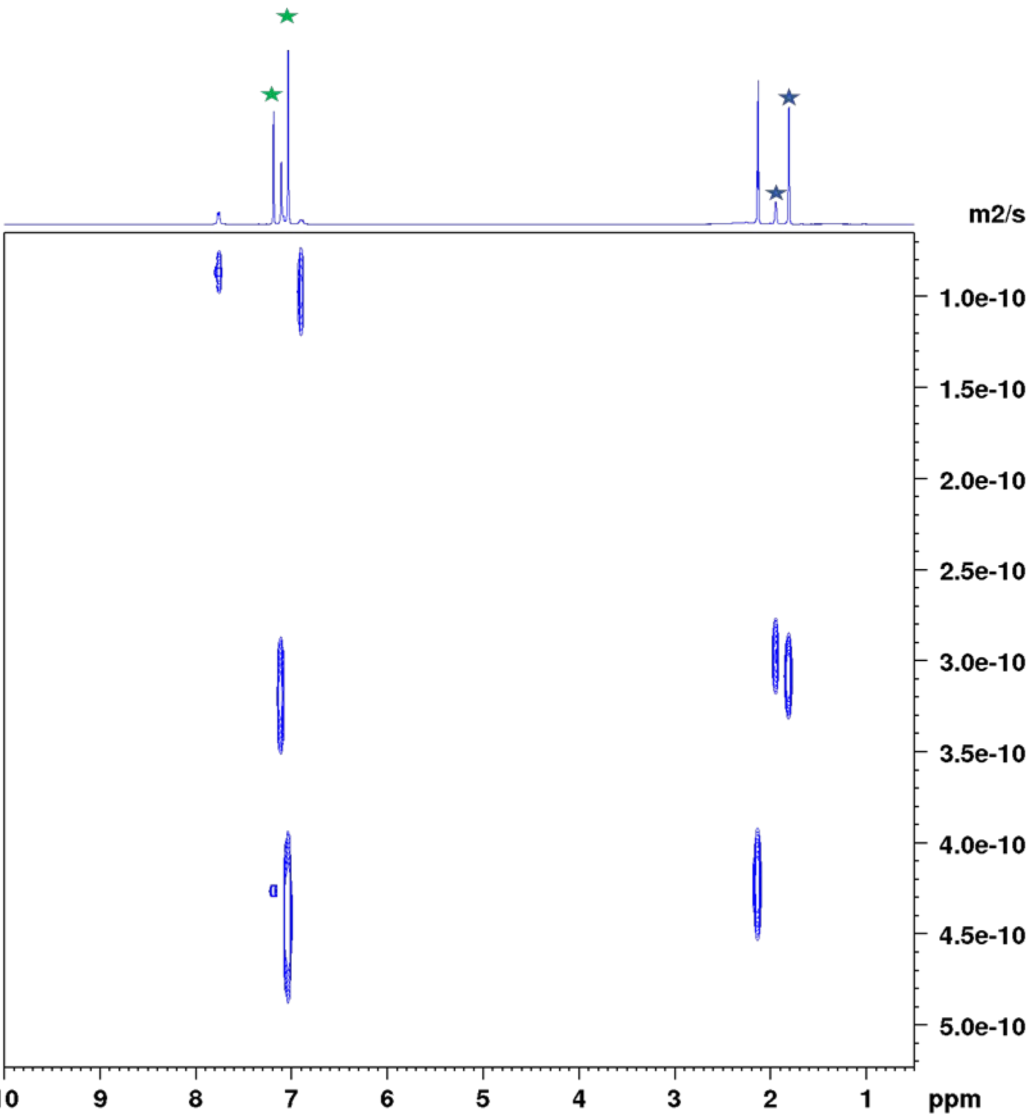

Figure S13: DOSY NMR spectrum of **5** in d_8-tol at 223 K. The DOSY acquisition parameters were adjusted, such that d20 = 0.1, to increase signal attenuation at this temperature. Residual protio solvent (green) and Adamantane (blue) (internal standard) are marked respectively.

Table S14: Diffusion data for compound **5** in d_8-tol at 223 K

	**Complex**	**Adamantane**
**Observed Diffusion Coefficient (m^2 s^{-1})**	8.21 x 10^{-11}	2.98 x 10^{-10}
**Normalised Diffusion Coefficient (m^2 s^{-1})**	3.94 x 10^{-10}	
**MW_{det} (g mol^{-1})**	1358	
**K (mol dm^{-3})**	7.74 x 10^{-8}	

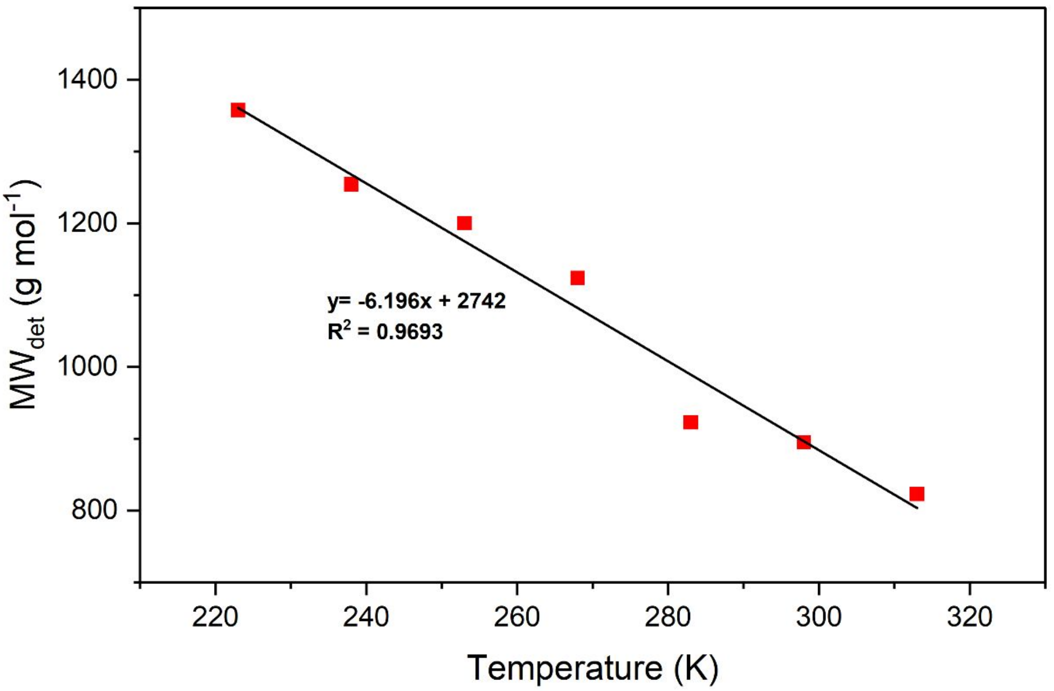

Figure S14: A plot of MW_{det} against temperature for compound **5**

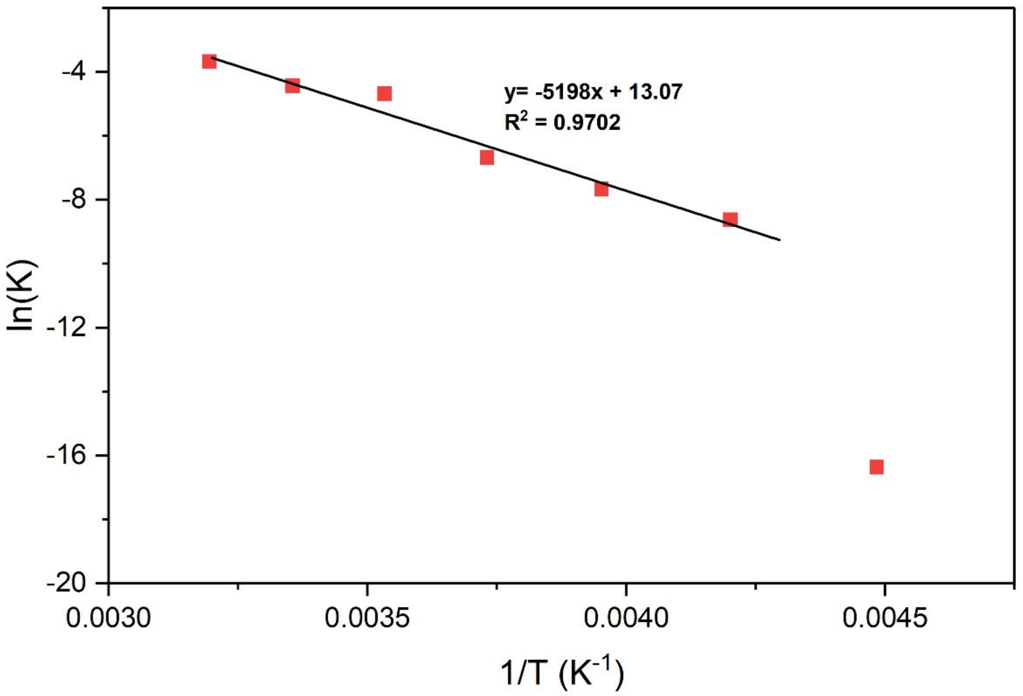

Figure S15: A plot of Ln(K) against 1/T for compound **5**, using equilibrium constants determined from DOSY NMR

Equilibria Calculations

Based on an equilibrium between the dimer, A and the monomer, B and assuming the observed diffusion coefficient is the weighted mean of the two components the equilibrium constant can be calculated as follows:

$$A \rightleftharpoons 2\mathrm{B}$$

$$Z = [A] + \frac{[B]}{2}$$

Where Z is the total sample concentration

The observed diffusion coefficient can be defined as follows:

$$D_{obs} = \frac{2[A]D_A + [B]D_B}{2[A] + [B]}$$

Where D_{obs} is the observed diffusion coefficient, and D_A and D_B are the calculated diffusion coefficients for the dimer and monomer respectively.

This can be rearranged by using the expression above for Z to give:

$$[A] = \frac{Z(D_{obs} - D_B)}{D_A - D_B}$$

Using the expression for Z, this allows the determination of $[A]$, and hence $[B]$ using the expression above. An equilibrium constant K can then be calculated.

$$K = \frac{[B]^2}{[A]}$$

Figure S15 can be used to estimate thermodynamic parameters for the monomer-dimer equilibrium as follows:

$$\ln K = -\frac{\Delta H}{RT} + \frac{\Delta S}{R}$$

As such, the gradient of Figure S15 can be used to determine ΔH,

$$\Delta H = -m \times R$$

With the intercept, c, then used to calculate ΔS,

$$\Delta S = R \times c$$

Linear regression of Fig S15 demonstrated the following:

Regression Statistics	
Multiple R	0.984976
R Square	0.970178

Adjusted R Square	0.962722
Standard Error	0.383666
Observations	6

	Coefficients	Standard Error	t Stat	P-value	Lower 95%	Upper 95%
Intercept	13.07245	1.675753439	7.800939	0.001456879	8.41981357	17.72509
X Variable 1	-5197.75	455.6493194	-11.4073	0.000336888	-6462.83056	-3932.66

Which therefore allowed the calculation of thermodynamic parameters for the monomer-dimer equilibrium as follows:

$$\Delta H = -43.2\ kJ\ mol^{-1}\ (\pm 3.79)$$

$$\Delta S = 109\ J\ K^{-1} mol^{-1}\ (\pm 13.9)$$

And given:

$$\Delta G = \Delta H - T\Delta S$$

$$\Delta G = 75.6\ kJ\ mol^{-1}\ (\pm 5.62)\ at\ 293K$$

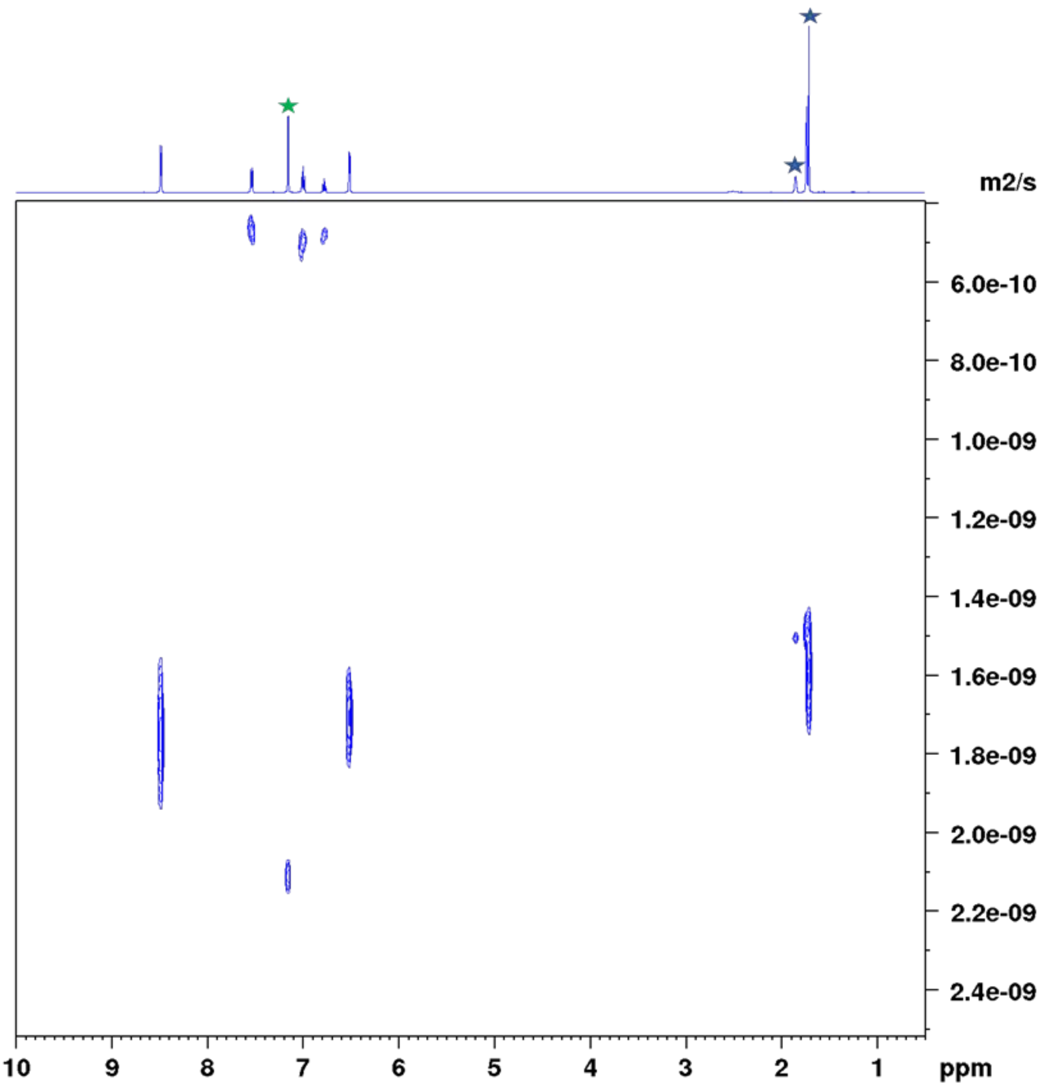

Figure S16: DOSY NMR spectrum of **3** in C_6D_6 with 4 excess equivalents of 4-Methyl pyridine at 298 K. Residual protio solvent (green) and Adamantane (blue) (internal standard) are marked respectively.

Table S15: Diffusion data for compound **3** in C_6D_6 with 4 excess equivalents of 4-Methyl pyridine

	**Phosphate**	**Base**	**Adamantane**
**Observed Diffusion Coefficient (m^2 s^{-1})**	4.40 x 10^{-10}	1.68 x 10^{-9}	1.47 x 10^{-9}
**Normalised Diffusion Coefficient (m^2 s^{-1})**	4.72 x 10^{-10}	1.80 x 10^{-9}	
**MW_{det} (g mol^{-1})**	1130	109	

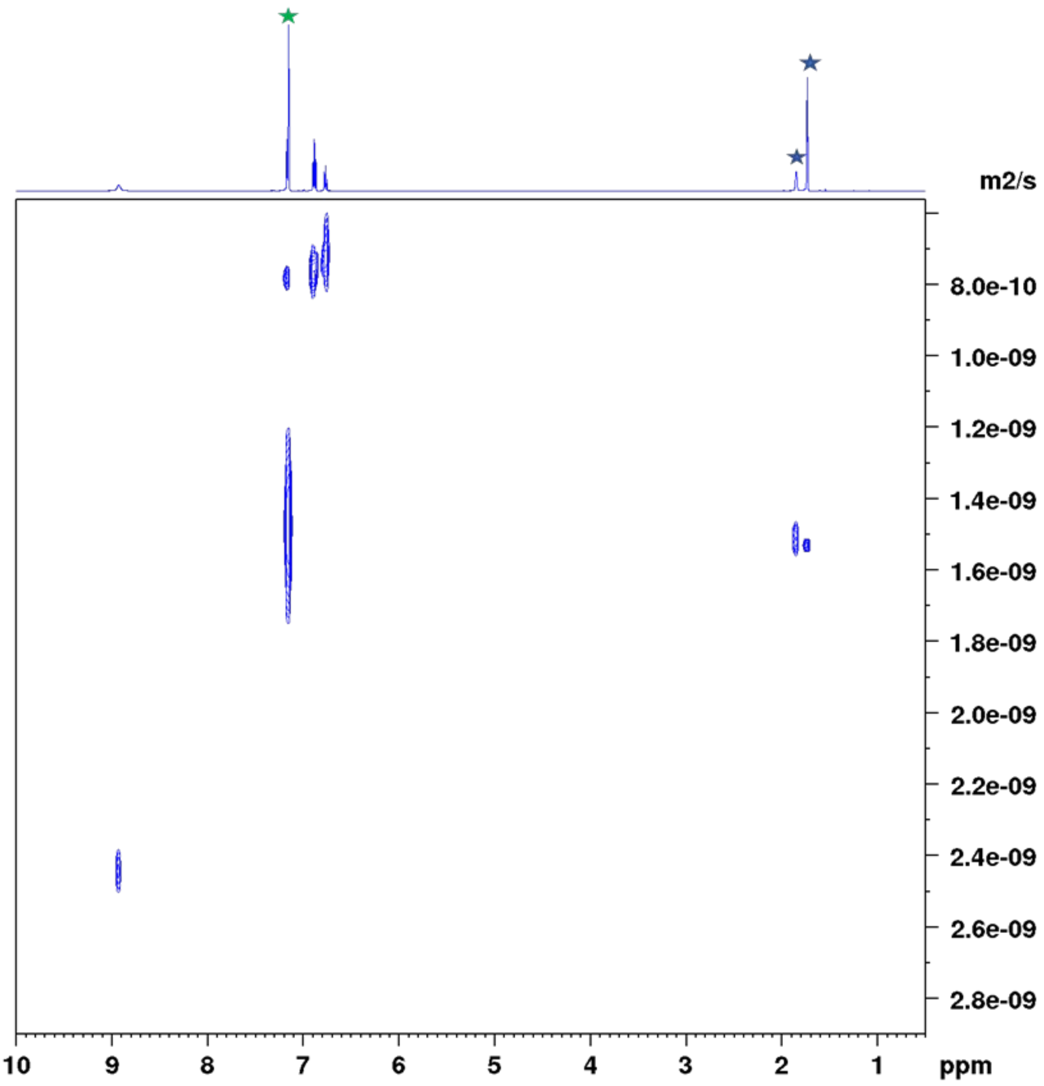

Figure S17: DOSY NMR spectrum of diphenylphosphoric acid in C_6D_6 at 298 K. Residual protio solvent (green) and Adamantane (blue) (internal standard) are marked respectively.

Table S16: Diffusion data for diphenylphosphoric acid

	**Compound**	**Adamantane**
**Observed Diffusion Coefficient (m^2 s^{-1})**	7.07×10^{-10}	1.50×10^{-9}
**Normalised Diffusion Coefficient (m^2 s^{-1})**	7.43×10^{-10}	
**MW_{cal} (g mol^{-1})**	500 [a]	
**MW_{det} (g mol^{-1})**	511	
**MW_{err}**	2 %	

[a] MW_{cal} assumes a dimeric structure of $[HO(O)P(OPh)_2]_2$

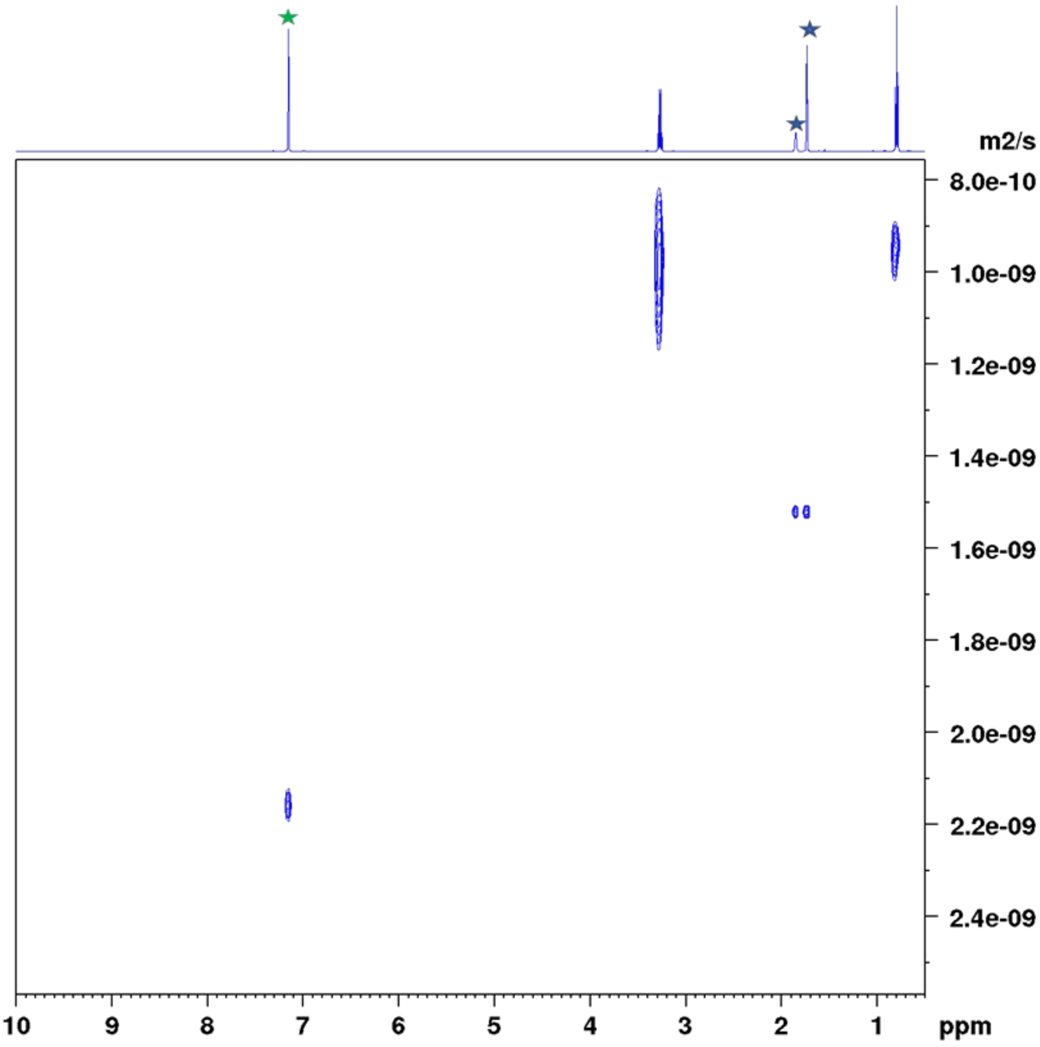

Figure S18: DOSY NMR spectrum of [Zn(S_2CNEt_2)] in C_6D_6 at 298 K. Residual protio solvent (green) and Adamantane (blue) (internal standard) are marked respectively.

Table S17: Diffusion data for [Zn(S_2CNEt_2)]

	**Compound**	**Adamantane**
**Observed Diffusion Coefficient (m^2 s^{-1})**	9.34 x 10^{-10}	1.50 x 10^{-9}
**Normalised Diffusion Coefficient (m^2 s^{-1})**	9.81 x 10^{-10}	
**MW_{cal} (g mol^{-1})**	362 [a]	
**MW_{det} (g mol^{-1})**	314	
**MW_{err}**	15 %	

[a] MW_{cal} assumes a monomeric structure of [Zn(S_2CNEt_2)$_2$]

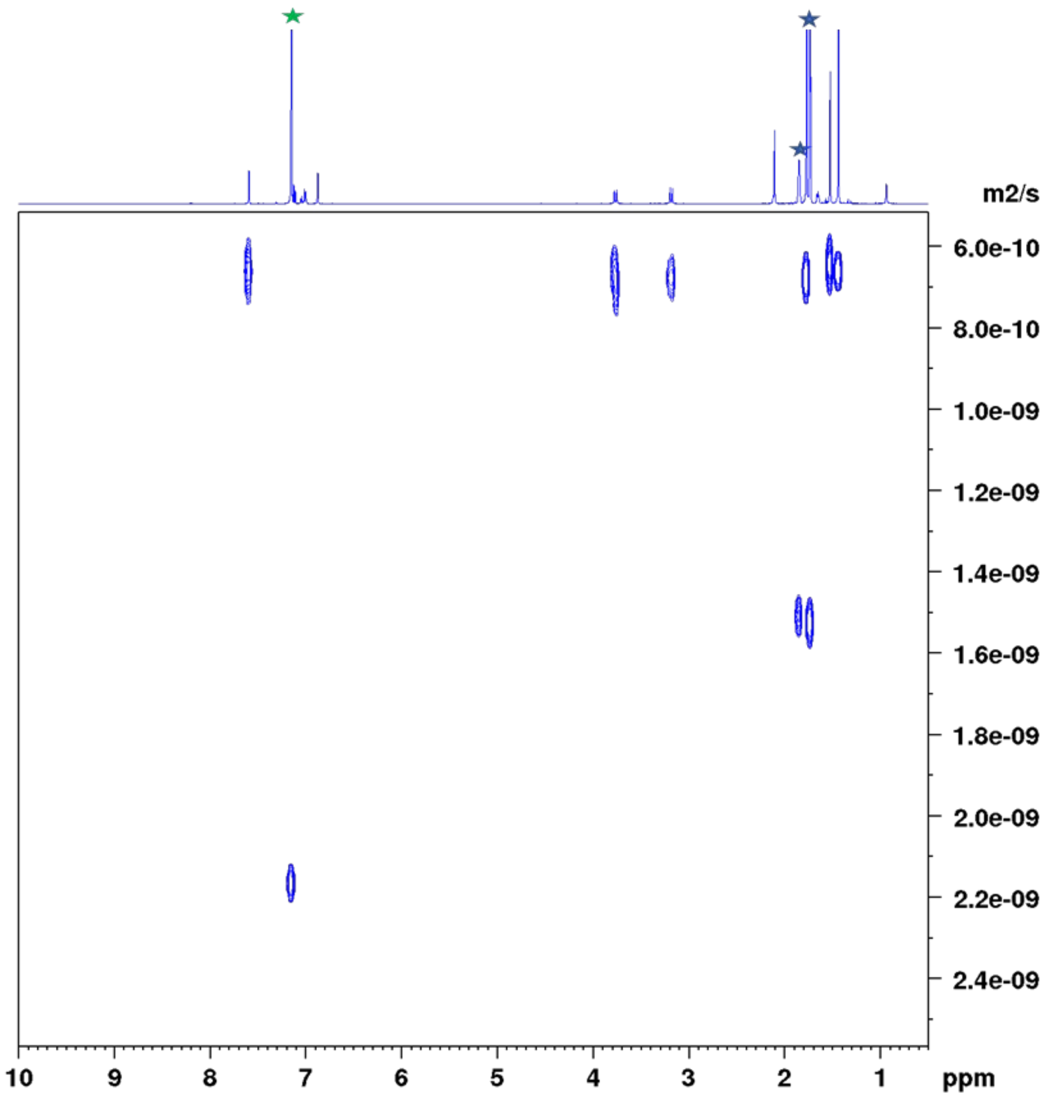

Figure S19: DOSY NMR spectrum of [Zn({$OC_6H_2{}^tBu_2CH_2$}$_2NC_2H_4NMe_2$)] in C_6D_6 at 298 K. Residual protio solvent (green) and Adamantane (blue) (internal standard) are marked respectively.

Table S18: Diffusion data for [Zn({$OC_6H_2{}^tBu_2CH_2$}$_2NC_2H_4NMe_2$)]

	**Compound**	**Adamantane**
**Observed Diffusion Coefficient (m^2 s^{-1})**	6.40 x 10^{-10}	1.48 x 10^{-9}
**Normalised Diffusion Coefficient (m^2 s^{-1})**	6.81 x 10^{-10}	
**MW_{cal} (g mol^{-1})**	588 [a]	
**MW_{det} (g mol^{-1})**	594	
**MW_{err}**	2 %	

[a] MW_{cal} assumes a monomeric structure as observed in the solid state.

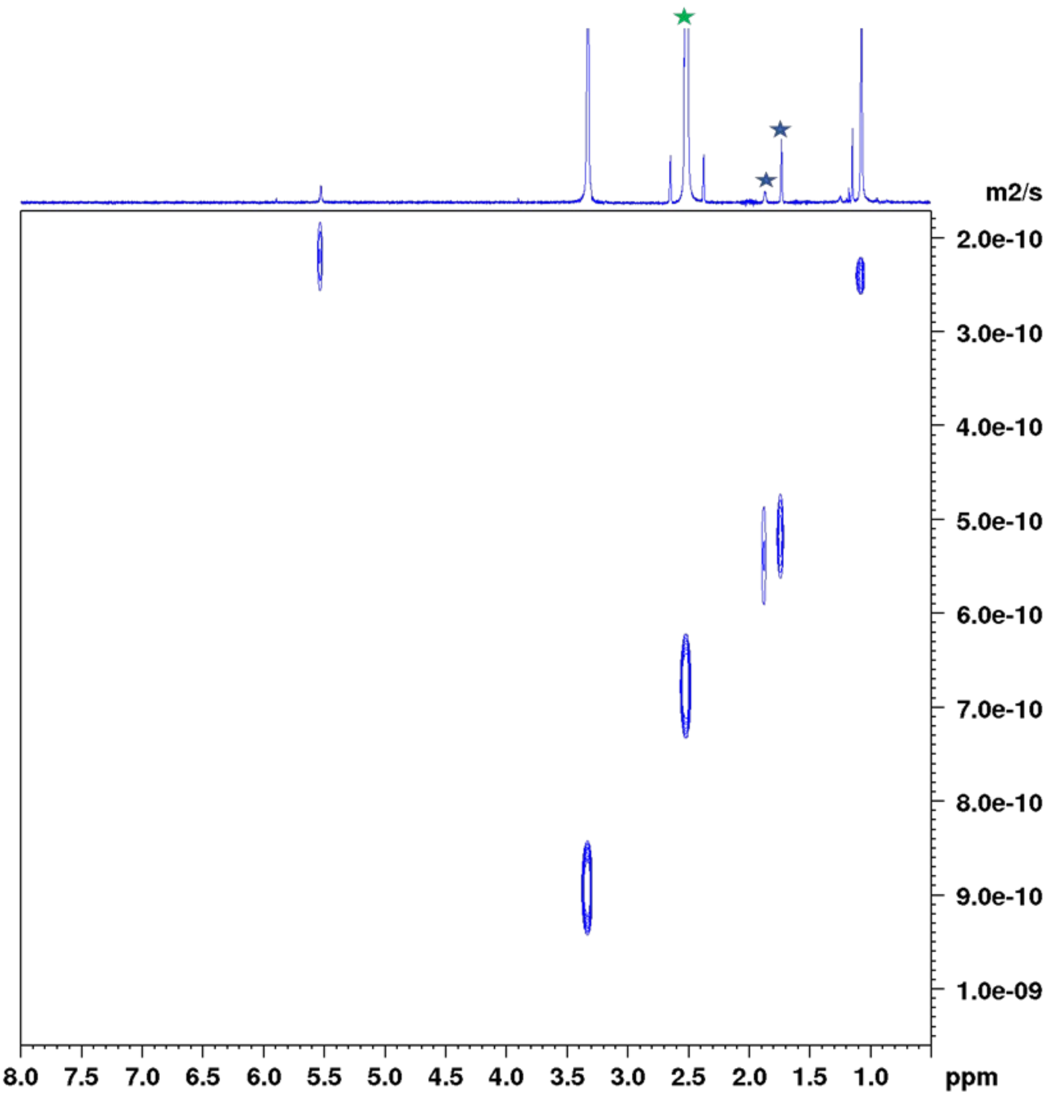

Figure S20: DOSY NMR spectrum of [Zn(2,2,6,6-tetramethyl-3,5-heptanedione)$_2$] in d6-DMSO at 298 K. Residual protio solvent (green) and Adamantane (blue) (internal standard) are marked respectively.

Table S19: Diffusion data for [Zn(2,2,6,6-tetramethyl-3,5-heptanedione)$_2$]

	**Compound**	**Adamantane**
**Observed Diffusion Coefficient (m^2 s^{-1})**	2.29 x 10^{-10}	5.05 x 10^{-10}
**Normalised Diffusion Coefficient (m^2 s^{-1})**	2.14 x 10^{-10}	
**MW_{cal} (g mol^{-1})**	432 [a]	
**MW_{det} (g mol^{-1})**	503	
**MW_{err}**	14 %	

[a] MW_{cal} assumes a monomeric structure as observed in the solid state

Supplementary References

1 H. S. Rathore, G. Varshney, S. C. Mojumdar and M. T. Saleh, Synthesis, characterization and fungicidal activity of zinc diethyldithiocarbamate and phosphate, *J. Therm. Anal. Calorim.*, 2007, **90**, 681–686.

2 Y. Sarazin, R. H. Howard, D. L. Hughes, S. M. Humphrey and M. Bochmann, Titanium, zinc and alkaline-earth metal complexes supported by bulky O, N, N, O-multidentate ligands: Syntheses, characterisation and activity in cyclic ester polymerisation, *Dalt. Trans.*, 2006, **60**, 340340–350350.

3 R. H. Howard, M. Bochmann and J. A. Wright, {Bis(3,5-Di-tert-butyl-2-oxidobenzyl)[2-(N,N-dimethylamino)ethyl] amine-κ4N,N′,O,O′}zinc(II) and {bis(3-tert-butyl-5- methyl-2-oxidobenzyl)-[2-(N,N-dimethylamino)ethyl]amine-κ4N, N′,O,O′}(tetrahdyrofuran)zinc(II), *Acta Crystallogr. Sect. C Cryst. Struct. Commun.*, , DOI:10.1107/S0108270106018695/FG3023SUP1.CIF.

4 N. S. Nandurkar, D. S. Patil and B. M. Bhanage, Ultrasound assisted synthesis of metal-1,3-diketonates, *Inorg. Chem. Commun.*, 2008, **11**, 733–736.
